# Efficacy and safety of early antibiotic de-escalation in febrile neutropenia for patients with hematologic malignancy: a systematic review and meta-analysis

**DOI:** 10.1128/aac.01597-24

**Published:** 2025-03-13

**Authors:** Yu-Han Chen, Andrea Yue-En Sun, Karishma Narain, Wei-Cheng Chang, Chieh Yang, Po-Huang Chen, Hong-Jie Jhou, Ming-Shen Dai, Natasha Rastogi, Cho-Hao Lee

**Affiliations:** 1 Department of Internal Medicine, Englewood Hospital and Medical Center 3224 , Englewood, New Jersey, USA; 2 College of Medicine, National Yang Ming Chiao Tung University, Taipei, Taiwan; 3 Department of Ophthalmology, Taoyuan General Hospital, Ministry of Health and Welfare 609994 , Taoyuan, Taiwan; 4 Jacob’s School of Medicine and Biomedical Sciences, State University of New York at Buffalo 12292 , Buffalo, New York, USA; 5 Division of Hematology and Oncology Medicine, Department of Internal Medicine, Tri-Service General Hospital, National Defense Medical Center, Taipei, Taiwan; 6 Department of Neurology, Changhua Christian Hospital 36596 , Changhua, Taiwan; Johns Hopkins University School of Medicine, Baltimore, Maryland, USA

**Keywords:** hematologic malignancies, febrile neutropenia, antibiotic de-escalation, broad-spectrum antibiotics, mortality reduction, hematopoietic stem cell transplantation

## Abstract

Febrile neutropenia (FN) is a serious complication in patients with hematologic malignancies following treatments such as chemotherapy and hematopoietic stem cell transplantation. It is typically managed with broad-spectrum antibiotics (BSA), but the optimal duration of BSA therapy remains controversial. This meta-analysis aimed to assess the clinical efficacy and safety of early antibiotic de-escalation in patients with hematologic malignancies with FN before hematopoietic recovery, compared to those who continued BSA until hematopoietic recovery. Statistical analysis included pooled odds ratios (OR) for mortality and secondary adverse outcomes, along with subgroup analysis to identify patient populations that may benefit from early de-escalation. Ten studies, mostly retrospective observational designs, were included. Early de-escalation significantly reduced mortality risk (OR 0.20, 95% CI 0.06–0.69). Subgroup analyses showed mortality benefits in older patients (>55 years old, OR 0.42, 95% CI 0.18–0.98) and in higher-quality studies (OR 0.07, 95% CI 0.01–0.62). No significant differences were observed for infection-related ICU admissions, bacteremia, recurrent fever, or *Clostridium difficile* infection (CDI). In conclusion, early de-escalation of BSA in patients with hematologic malignancies and developing FN after treatment significantly reduces mortality risk without increasing major adverse events. These findings support the use of early de-escalation and highlight the need for personalized strategies to improve patient outcomes.

## INTRODUCTION

High-risk patients with malignancies who undergo treatments such as chemotherapy or hematopoietic stem cell transplantation (HSCT) often experience neutropenia, resulting in increased rates of infection (1–4). The immunocompromised status of patients with hematologic malignancies puts them at risk of infection. Additionally, infection remains one of the major causes of morbidity and mortality in patients who have undergone treatments such as chemotherapy and HSCT (3, 5–7).

There is currently no consensus on the duration of broad-spectrum antibiotic (BSA) use against febrile neutropenia (FN). The guidelines from the Infectious Diseases Society of America (IDSA) in 2011 recommend maintaining the initial antibiotic regimen in neutropenic patients with unexplained fever until the absolute neutrophil count (ANC) exceeds 500 cells/µL, regardless of the clinical course or identification of infection (8). The American Society of Clinical Oncology (ASCO) and European Society for Medical Oncology (ESMO) update that in high-risk patients, the course of antibiotics should be completed according to the identified infection source or continued until the ANC exceeds 500 cells/µL (9, 10). The National Comprehensive Cancer Network (NCCN) guidelines suggest that the duration of antibiotic use depends on several factors, including the source of infection, clinical course, neutropenia recovery, toxicity, and the opinions of infectious disease consultants (11). In contrast, guidelines from the European Conference on Infections in Leukemia (ECIL) propose that empiric antibiotics can be stopped after 72 hours in patients proven to be hemodynamically stable and afebrile for at least 48 hours, regardless of ANC (12, 13).

However, extensive utilization of BSA has been shown to increase the likelihood of contracting multidrug-resistant organisms and invasive fungal infections. These multidrug-resistant organisms can increase patient morbidity and mortality, prolong hospital stays, and escalate healthcare costs, thus posing an emerging threat to patient care (14–16). The average duration of hematopoietic recovery varies depending on the severity of neutropenia and individual patient conditions but is generally around 2 weeks (17, 18). It is therefore imperative to practice antibiotic stewardship and examine whether de-escalation before complete hematopoietic recovery is a viable strategy. Previous systematic review has indicated that early de-escalation before hematopoietic recovery in HSCT patients is feasible, but most studies were small (19). Due to limited clinical data on the feasibility and safety of de-escalation antibiotic therapy, we conducted a systematic review and meta-analysis of existing studies to compare the clinical efficacy and safety of antibiotic de-escalation in patients with hematologic malignancies who underwent treatment and developed FN before hematopoietic recovery versus those who continued BSA until clinical signs of hematopoietic recovery.

## MATERIALS AND METHODS

### Search strategy

A thorough review of existing literature was conducted to locate all historical studies published up to January 2024. Primary sources included the PubMed, EMBASE, Cochrane Library, and Clinical Trials databases. Electronic searches were carried out using a search algorithm that combined MeSH terms, Emtree synonyms, and free words. The search strategy was designed to be comprehensive and sensitive, ensuring the identification of all relevant studies comparing early de-escalation of BSA with standard use of BSA in hematologic cancer patients with FN.

In addition to the electronic database searches, supplementary search methods were employed to minimize the risk of missing important studies. These methods included hand searching the reference lists of included studies and relevant review articles, as well as searching for gray literature sources such as conference proceedings, abstracts, and unpublished trials. Experts in the field were also consulted to identify any ongoing or recently completed studies that may not have been captured by our search strategy. There were no restrictions on the publication language of the studies, and translations were obtained when necessary.

The detailed search strategy, including the specific MeSH terms, Emtree synonyms, and free words used, is provided in the supplemental material (Supplement 1). The search results were independently screened by two reviewers (C.H.L. and W.C.C.) to identify potentially eligible studies based on the title and abstract. The full texts of these studies were then retrieved and assessed for inclusion based on the predefined eligibility criteria.

### Inclusion and exclusion criteria

The Preferred Reporting Items for Systematic Reviews and Meta-Analyses (PRISMA) checklist was used to guide the reporting of this meta-analysis (20). The inclusion criteria for this meta-analysis were as follows: studies involving patients diagnosed with hematologic malignancies post-treatment, such as chemotherapy or bone marrow transplant, with a definite diagnosis of neutropenia (ANC lower than 500 cells/mm^3^ or expected to decrease to lower than 500 cells/mm^3^ within 48 hours) and a febrile condition (single temperature of 38.3°C [101°F] or a temperature of 38.0°C [100.4°F] sustained over a 1 hour period). Interventions investigated include antimicrobial stewardship practices, particularly the de-escalation or discontinuation of antibiotics in patients with FN. The outcomes reported should encompass clinical outcomes such as the incidence of mortality, recurrent fever, major infection rates, ICU transfer rates, and adverse events, along with evaluations of the impact of antibiotic de-escalation on patient health and recovery. The study design includes retrospective and prospective cohort studies and randomized controlled trials (RCTs). All articles that met the inclusion criteria were included up to January 2024.

The exclusion criteria for this study were as follows: studies that do not specifically address FN in the context of hematologic malignancies, studies involving chronic neutropenia or congenital neutropenia populations, articles that do not specifically focus on antibiotic stewardship or de-escalation strategies, and research that discusses general antimicrobial use without specific reference to FN. Additionally, non-research articles such as case reports, editorials, reviews, and commentaries were excluded from the analysis. In cases of multiple publications from the same study population, only the most recent or comprehensive report was included to avoid duplication of data. If necessary, the authors of the original studies were contacted for clarification or to obtain additional data.

### Data extraction

Two reviewers (C.-H.L. and W.-C.C.) independently extracted data from the included studies using a standardized data collection form. The extracted information included study characteristics (first author, year of publication, study design, country, sample size, inclusion, and exclusion criteria), patient characteristics (study population, age, duration of hospital stay, duration of neutropenia, patient underlying hematologic diseases, and treatment received), treatment details (time of early de-escalation, de-escalation intervention details), outcomes (mortality, infection-related ICU admission, bacteremia, recurrent fever, Clostridium difficile infection [CDI]), and funding sources.

Any discrepancies in the extracted data between the two reviewers were resolved through discussion and consensus. If a consensus could not be reached, a third reviewer (Y.-H.C.) was consulted to make the final decision. The extracted data were then entered into a Microsoft Excel spreadsheet for further analysis.

### Quality assessment

The quality of the included studies was independently assessed by two reviewers (C.-H.L. and A.Y.-E.S.) using RONBIS I in non-randomized studies and Cochrane Risk of Bias Assessment in randomized studies (21, 22). Any discrepancies in the quality assessment between the two reviewers were resolved through discussion and consensus. If a consensus could not be reached, a third reviewer (Y.H.C.) was consulted to make the final decision.

### Statistical analysis

The extracted data were analyzed using R (Version 2024.04.2+764 by Posit Software, PBC). The primary outcomes of interest were mortality, and secondary outcomes were adverse events such as infection-related ICU admission, bacteremia, recurrent fever, and CDI. Odds ratio (OR) of mortality, infection-related ICU admission, bacteremia, recurrent fever, and CDI and their corresponding 95% confidence intervals (CIs) were extracted from the original studies. ORs and their 95% CIs were calculated based on the number of events and total number of patients in each group.

The ORs from individual studies were then pooled using a random-effects model and a common/fixed-effect model (23). The random-effects model was chosen over a common/fixed-effects model because it provides a more conservative estimate of the treatment effect and is more appropriate when heterogeneity is expected among the included studies (24).

All statistical tests were two-sided, and a *P*-value < 0.05 was considered statistically significant, except for the heterogeneity tests, where a *P*-value < 0.10 was used. The results of the meta-analysis were presented using forest plots, which display the effect estimates and 95% CIs for each individual study, as well as the pooled effect estimate. The forest plots also include the weight assigned to each study based on its sample size and the precision of its effect estimate.

Heterogeneity among the studies was assessed using the Cochrane Q test and quantified using the I^2^ statistic, which represents the percentage of total variation across studies that is due to heterogeneity rather than chance (24, 25). An I^2^ value of 25% was considered to indicate low heterogeneity, 50% was considered to indicate moderate heterogeneity, and 75% was considered to indicate high heterogeneity. If significant heterogeneity was observed (I^2^ > 50% or *P* < 0.10 for the Q test), meta-regression and subgroup analyses were done to explore potential sources of heterogeneity, such as patient characteristics, treatment regimens, and study design.

Sensitivity analyses were performed to assess the robustness of the meta-analysis results by excluding studies with a high risk of bias or studies with outlying results. Trial sequential analysis (TSA) was also applied to examine the risks of type I and type II errors and to determine the required information size (RIS) for a conclusive meta-analysis (26).

Publication bias was evaluated visually using funnel plots and statistically using Egger’s regression test (27). The funnel plots of the primary and secondary outcomes were included in the supplemental material (Supplements 2–6). A symmetric funnel plot and a non-significant Egger’s test (*P* > 0.05) were considered to indicate the absence of publication bias, while an asymmetric funnel plot and a significant Egger’s test were considered to indicate the presence of publication bias. If publication bias was detected, the trim-and-fill method was used to estimate the impact of potential missing studies on the pooled effect size (28).

## RESULTS

### Patient characteristics

**Fig 1:**
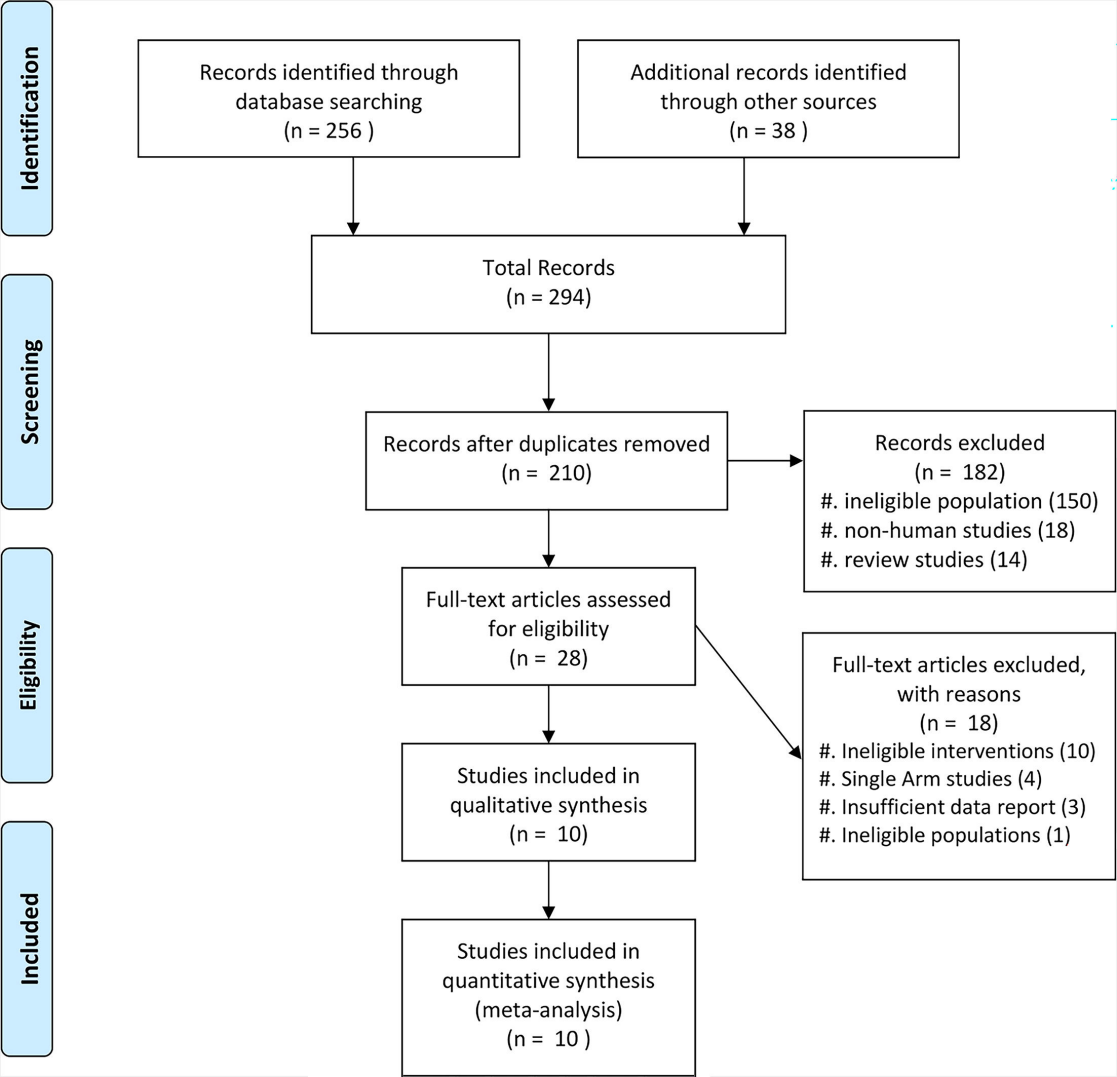
PRISMA flow diagram.

Ten studies were included in this review, with details listed in Table 1 (29–38). The selection of the articles is summarized in Fig. 1. The results of the quality assessment for each included study are presented in Tables 2 and 3. The studies were predominantly retrospective observational designs, with one interventional study, one quasi-experimental pre-post study, and one RCT. These studies were conducted across various countries, including Belgium, Italy, India, Spain, and the United States. The patient populations included adult patients with hematological malignancies such as acute myeloid leukemia (AML), acute lymphoblastic leukemia (ALL), multiple myeloma (MM), lymphoma, and other hematological malignancies, with AML being the most prevalent diagnosis in most studies. Patients received either HSCT or chemotherapy, and most of them developed FN of unknown origin during their treatment course. One study had a patient with carbapenem-resistant Enterobacteriaceae bacteremia (blood culture positive without polymicrobial growth) and febrile neutropenia (36). The median age of patients ranged from 49 to 63 years across the studies. The duration of hospitalization varied considerably, with reported medians spanning from 20 to 32 days, while the duration of neutropenia ranged from 2 to 25 days (median values). Sample sizes varied widely, from small cohorts of 44 patients to larger studies including up to 958 participants.

**TABLE 1:** Basic characteristics of included studies^*a*^

	Design (country)	Study target	Early de-escalation definition (within *x* hours)	Intervention (E)	Comparison (C)	Number of patients (E/C)^*c*^	Median age (E/C)	Duration of hospitalization in days (E/C)	Duration of neutropenia in days (E/C)	Number of patients who received allogenic transplants/autologous transplant/chemotherapy/CAR-T		Number of patients with underlying AML/ALL/MM/lymphoma/other^*d*^	
										E	C	E	C
Aguilar-Guisado (38)	Open-label, randomized, controlled phase 4 trial (Spain)	Adults with hematological malignancies or undergoing HSCT with high-risk febrile neutropenia	72	De-escalation from empirical antibiotics, which are based on antipseudomonal beta-lactam drug as monotherapy (ceftazidime, cefepime, meropenem, imipenem, or piperacillin-tazobactam) or as combination therapy with an aminoglycoside, fluoroquinolone, or glycopeptide	Until hematopoietic recovery	78/79	52/54	NA/NA	14/11	9/29/39/0	5/43/31/0	40 (study listed only acute leukemia)/7/23/8	31 (study listed only acute leukemia)/14/29/5
Snyder (32)	Retrospective observational study (US)	Adult allo-HSCT recipients that developed FN of unknown origin	120	De-escalation from an antipseudomonal beta-lactam (aztreonam, cefepime, ceftazidime, meropenem, piperacillin, tazobactam) alone or in combination with an agent with expanded Gram-positive coverage (vancomycin, daptomycin, and linezolid) and/or an aminoglycoside (tobramycin) to original prophylactic antimicrobial agent	Until hematopoietic recovery^*b*^	46/74	58/57	20/20	2/3	46/0/0/0	74/0/0/0	14/5/0/4/23	36/9/0/9/20
Gustinetti (37)	Retrospective observational study (Italy)	Adult allogeneic HSCT recipients with FN of unknown origin	96	De-escalation (within 96 hours from onset of first antibiotic treatment) from piperacillin-tazobactam or meropenem to a narrower spectrum beta-lactam or stopping any antibiotic OR discontinuing therapy by stopping empirical therapy and then resuming fluoroquinolone prophylaxis	De-escalation at any time before engraftment	26/57	49/49	NA/NA	17/17	26/0/0/0	57/0/0/0	18/0/0/0/8	33/0/0/0/24
Petteys (29)	Retrospective study of adult HCT recipients (US)	Adult HSCT recipients with FN of unknown origin	NA	De-escalation from broad spectrum antibiotics with anti-pseudomonal beta lactam (cefepime, piperacillin-tazobactam, meropenem), or aztreonam if drug allergy, with expanded Gram-positive coverage (vancomycin, daptomycin or linezolid) and/or aminoglycoside (tobramycin) or fluoroquinolone (ciprofloxacin)	Until hematopoietic recovery	24/83	62.5/60	28/21	15/4	23/1/0/0	13/70/0/0	21 (study listed only acute leukemia)/2/1/0	9 (study listed only acute leukemia)/42/32/0
Fuller (30)	Retrospective chart review (US)	Adult patients with AML undergoing induction chemotherapy who develop FN of unknown origin	NA	De-escalation from broad spectrum intravenous therapy (piperacillin-tazobactam, cefepime, carbapenems, or aztreonam) to either prophylactic levofloxacin or cessation of antibiotics	Until hematopoietic recovery	38/39	NA/NA	NA/NA	NA/NA	0/0/38/0	0/0/39/0	38/0/0/0	39/0/0/0
Rearigh (31)	Retrospective study (US)	Adult patients who received autologous or allogeneic hematopoietic stem cell transplant with FN	48	De-escalation from monotherapy with an antipseudomonal beta-lactam (most commonly cefepime or piperacillin-tazobactam) to fluoroquinolone prophylaxis	Until hematopoietic recovery	83/214	53.7/56.8	NA/NA	9.1/8	36/47/0/0	31/183/0/0	13/9/8/40/13	11/3/80/102/18
Verlinden (33)	Interventional study without concurrent controls (Belgium)	Adult patients who received chemotherapy or HSCT with febrile neutropenia and without documented infection	72	De-escalation from meropenem and amikacin combination therapy to meropenem monotherapy after 3 instead of 5 days (in the absence of MDR strains)	De-escalation after 5 days	446/512	59/58	27/27	15/15	123/134/189/0	137/143/232/0	217/31/82/41/75	223/42/96/51/100
Ly (34)	Retrospective, single-center, observational cohort (US)	Adult patients receiving chemotherapy for AML or ALL with febrile neutropenia of unknown origin	168	De-escalation from antipseudomonal antibiotic (cefepime, meropenem, piperacillin-tazobactam, aztreonam, etc.) to prophylaxis (oral levofloxacin) for patients who meet the following criteria: (i) received at least 7 days of guideline-based beta-lactam antibiotic with anti-pseudomonas activity and (ii) achieved clinical recovery with each of the following in the last 72 hours: afebrile, hemodynamic stability, stable physical exam imaging not suggesting infection	Until hematopoietic recovery	19/25	49/51	30/32	23/25	0/0/19/0	0/0/25/0	8/11/0/0/0	23/2/0/0/0
Alegria (35)	Pre–post quasi-experimental study (US)	Adult patients who are receiving high-intensity induction chemotherapy for newly diagnosed or relapsed/refractory AML with FN of unknown origin	120	De-escalation from antipseudomonal b-lactam (piperacillin/tazobactam, cefepime, or meropenem) to levofloxacin for patients with relapsed/refractory AML or de-escalation to cessation of antibiotics for patients with newly diagnosed AML	Until hematopoietic recovery	53/40	63/63	27/29	NA/NA	0/0/53/0	0/0/40/0	53/0/0/0/0	40/0/0/0/0
Bansal (36)	Retrospective study (India)	Adult hematology-oncology patients with carbapenem-resistant Enterobacteriaceae bacteremia (blood culture positive without poly-microbial growth) and febrile neutropenia	72	De-escalation from CRE drugs, if started (e.g., colistin, fosfomycin, tigecycline, minocycline, ceftazidime-avibactam; either alone or in combination) to cessation of antibiotics within 72 hours	Continuation of CRE drugs for more than 72 hours	97/42	42/41	NA/NA	19/24	19 (autologous and allogenic HSCT)/NA/NA	27 (autologous and allogenic HSCT)/NA/NA	69/NA/8/6/NA	23/NA/4/6/NA

^*a*^ NA, not reported.

^*b*^ Hematopoietic recovery: first day of absolute neutrophil count 500 cells/mm^3^ for three consecutive days.

^*c*^ E/C is the number of patients in the experiment group (E)/number of patients in the control group (C).

^*d*^ AML, acute myeloid leukemia; ALL, acute lymphoblastic leukemia; MM, multiple myeloma.

**TABLE 2:** ROBINS-I assessment for risk of bias^*a*^

Study author (ref. no.)	Bias due to confounding	Bias in selection of participants for the study	Bias in classification of interventions	Bias due to deviations from intended interventions	Bias due to missing data	Bias in measurement of the outcome	Bias in selection of the reported result	Overall bias
Snyder (32)	S	M	L	L	L	L	M	S
Gustinetti (37)	S	M	L	M	L	L	M	S
Petteys (29)	S	S	L	L	L	M	M	S
Fuller (30)	M	M	L	L	L	L	M	M
Rearigh (31)	M	M	L	L	L	L	M	M
Verlinden (33)	S	M	L	L	L	L	M	S
Ly (34)	M	L	L	L	M	L	M	M
Alegria (35)	M	L	L	L	L	L	M	M
Bansal (36)	M	S	L	L	L	L	M	S

^*a*^ L, low risk; M, moderate risk; S, serious risk.

**TABLE 3:** Cochrane risk of bias assessment for randomized control trials^*a*^

Study author (ref. no.)	Random sequence generation	Allocation concealment	Blinding of participant and personnel	Blinding of outcome assessment (subjective)	Blinding of outcome assessment (objective)	Incomplete outcome data	Selective reporting	Other bias
Aguilar-Guisado (38)	L	L	H	H	L	L	L	L

^*a*^ L, low risk; U, unclear risk; H, high risk.

The early de-escalation approaches included switching from antipseudomonal beta-lactams such as cefepime, piperacillin-tazobactam, or meropenem to prophylactic fluoroquinolones (often levofloxacin), discontinuing combination therapies in favor of monotherapy, transitioning from carbapenems to narrower-spectrum beta-lactams, or ceasing all antimicrobial therapy. The timing of de-escalation varied among studies, with most implementing the strategy within 2–5 days of initial treatment, while the comparison groups continued the BSA until hematopoietic recovery (Table 1).

### Mortality

**Fig 2:**
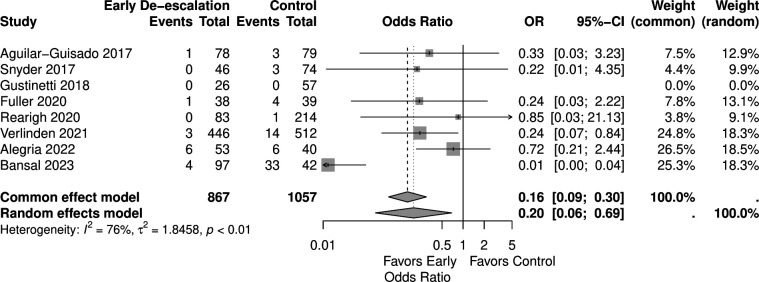
Forest plot of mortality.

**Fig 3:**
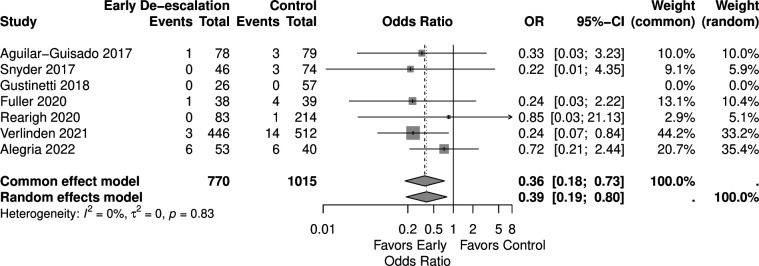
Forest plot of mortality sensitivity test.

**Fig 4:**
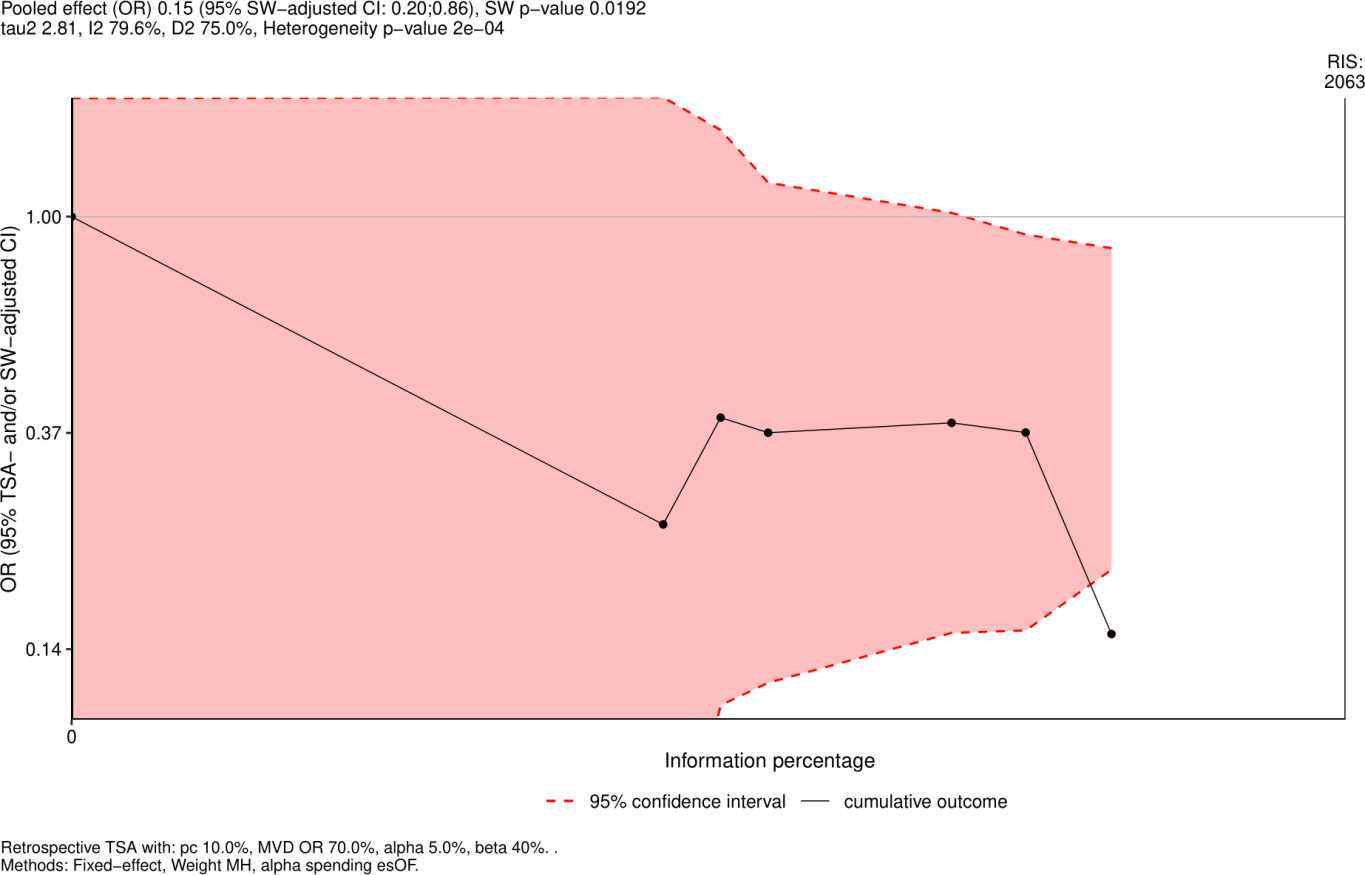
TSA of mortality.

Eight studies reported outcomes of mortality that were included in the analysis with 867 patients in the early de-escalation group and 1,057 patients in the control group. The Aguilar-Guisado 2017 study monitored mortality for 28 days (38), Gustinetti 2018 for 60 days (37), and Rearigh 2020 for 30 days (31). Snyder 2017 (32), Fuller 2020 (30), Rearigh 2020 (31), Verlinden 2021 (33), and Bansal 2023 (36) were retrospective studies and evaluated mortality during hospitalization. The result revealed that early de-escalation had a significantly lower mortality risk with an OR of 0.20 and 95% CI of 0.06–0.69 (Fig. 2). However, there was significant heterogeneity observed between the included studies (I^²^ = 76%, *P* < 0.01). A sensitivity test was done excluding Bansal 2023 study (36), as its mortality outcome was identified as a statistical outlier. This study reported significantly different mortality rates compared to the other included studies, contributing to increased heterogeneity. The result remaining significantly lower mortality risk with an OR of 0.39 and a 95% CI of 0.19–0.80. I^²^ was 0% with low heterogeneity (Fig. 3). A TSA was also conducted to assess the effect of early de-escalation on mortality risk, while Bansal et al. reported only all-cause mortality, which might lead to over- or under-estimated mortality in patients with hematologic malignancies, which revealed a significant protective effect of the intervention before reaching the RIS of 2,063 participants (Fig. 4). The cumulative Z-curve crossed the lower boundary of the trial sequential monitoring boundaries, yielding a pooled OR of 0.15 (95% CI: 0.20–0.86, *P* = 0.02).

### Secondary outcome

**Fig 5:**
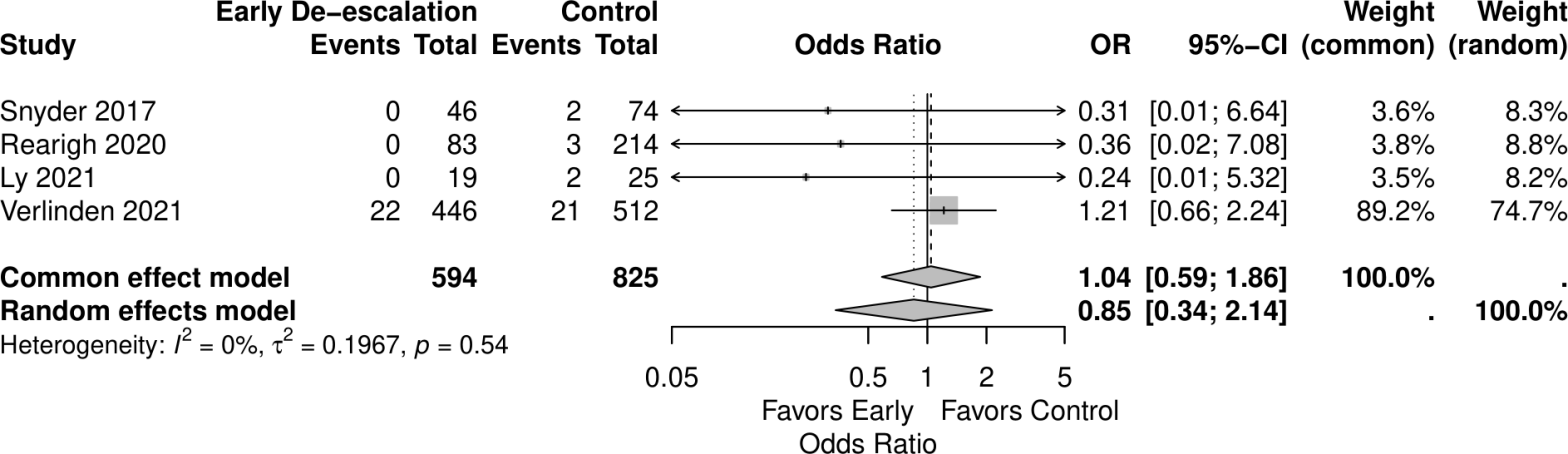
Forest plot of infection-related ICU admissions.

Four studies reported infection-related ICU admissions involving 1,419 patients (594 in the early de-escalation group and 825 in the control group). Early de-escalation was not associated with a significant difference in the risk of infection-related ICU admission compared to control (OR 0.85, 95% CI 0.34–2.14) (Fig. 5).

**Fig 6:**
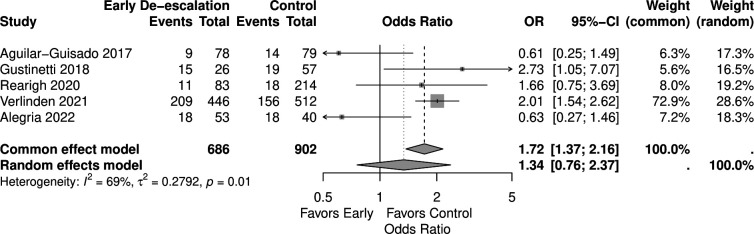
Forest plot of bacteremia.

In the analysis for bacteremia, which included five studies with 1,588 patients (686 in the early de-escalation group and 902 in the control group), the random-effects model showed no significant difference (OR 1.34, 95% CI 0.76–2.37). However, the common-effects model indicated a significantly higher risk of bacteremia compared to control (OR 1.72, 95% CI 1.37–2.16) (Fig. 6).

**Fig 7:**
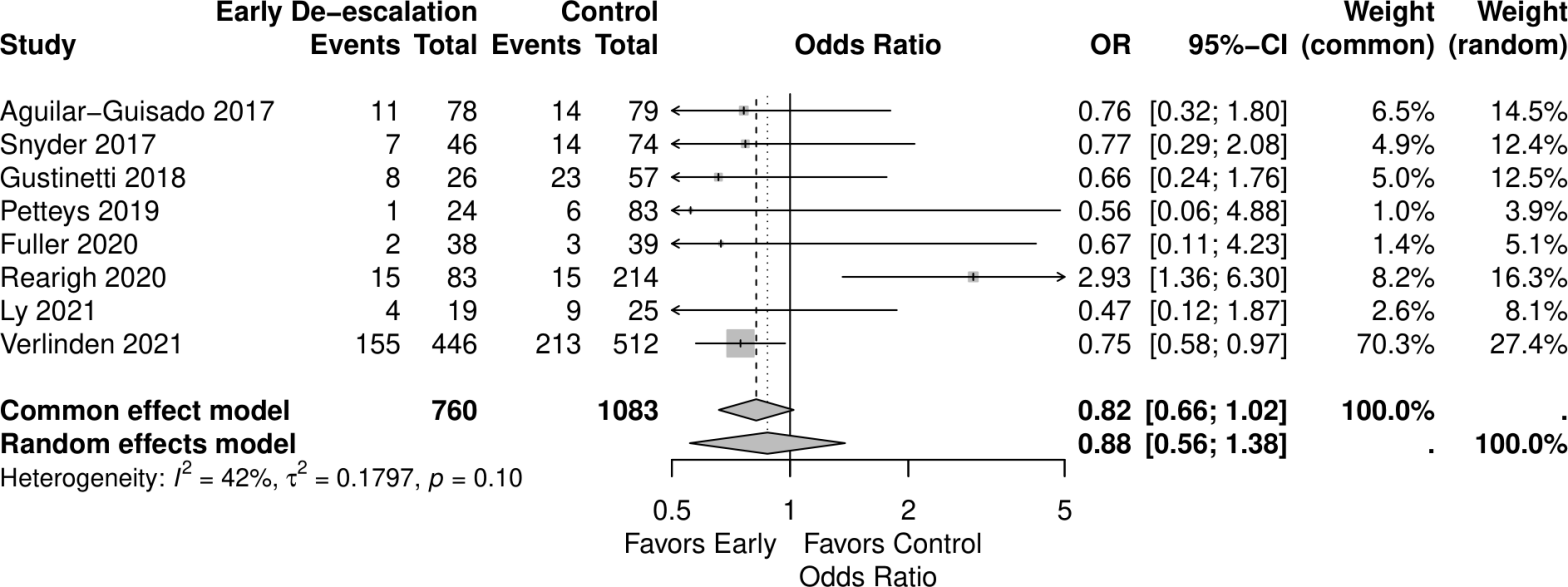
Forest plot of recurrent fever.

Regarding recurrent fever, eight studies reported the outcome involving 1,843 patients (760 in the early de-escalation group and 1,083 in the control group). Early de-escalation was not associated with a significant difference in the risk of recurrent fever compared to the control group (OR 0.88, 95% CI 0.56–1.38) (Fig. 7).

**Fig 8:**
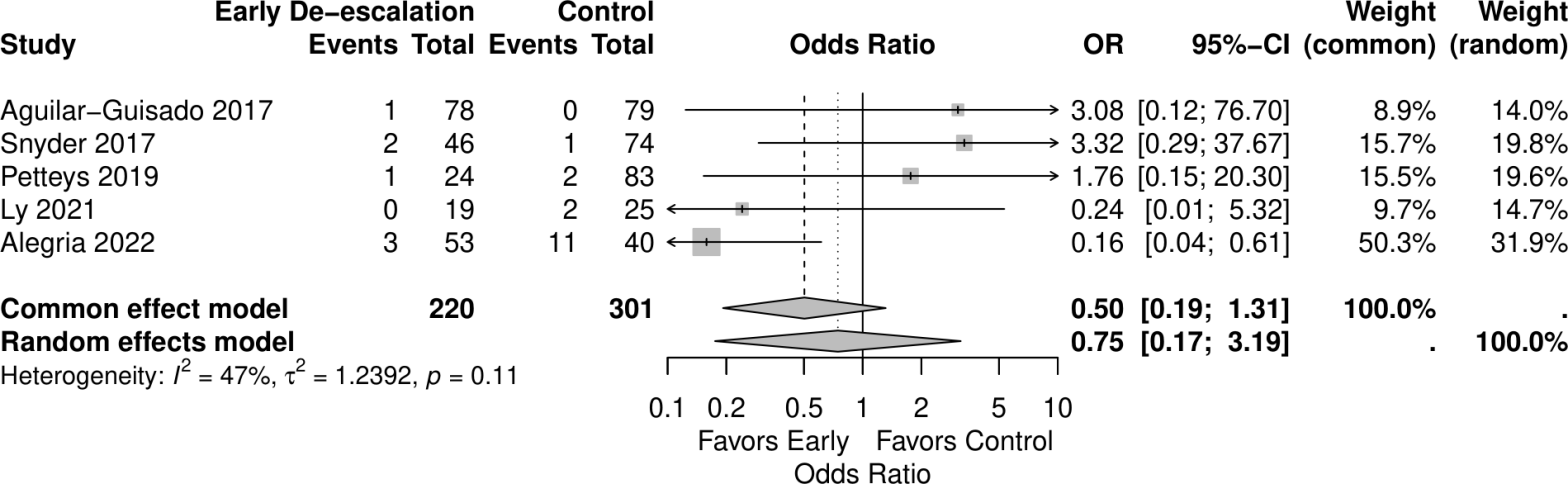
Forest plot of CDI.

In the analysis of CDI outcome, which included five studies with 521 patients (220 in the early de-escalation group and 301 in the control group), early de-escalation was associated with a non-significant reduction in the risk of CDI compared to control (OR 0.75, 95% CI 0.17–3.19) (Fig. 8).

### Subgroup analysis

To explore potential sources of heterogeneity and identify subgroups that may derive greater benefit from early de-escalation, several subgroup analyses were performed on the primary outcome of mortality, including the proportion of patients with AML, the timing of de-escalation, median age, duration of neutropenia, studies quality, and studies published year.

**Fig 9:**
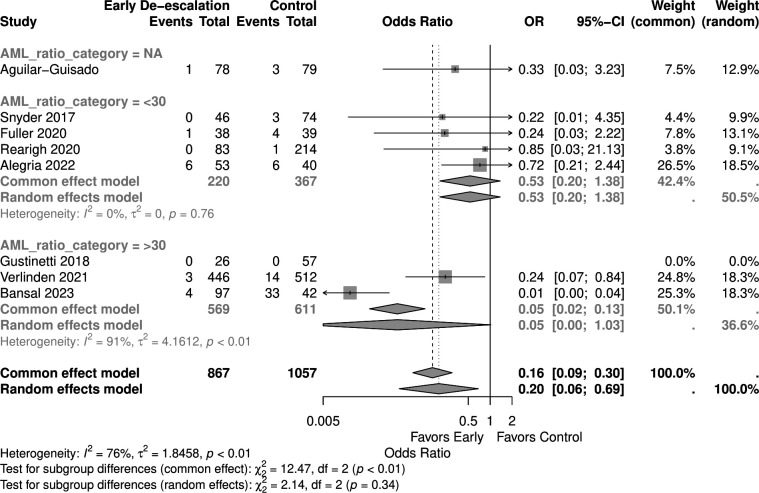
Forest plot of mortality subgroup analysis of AML ratio.

**Fig 10:**
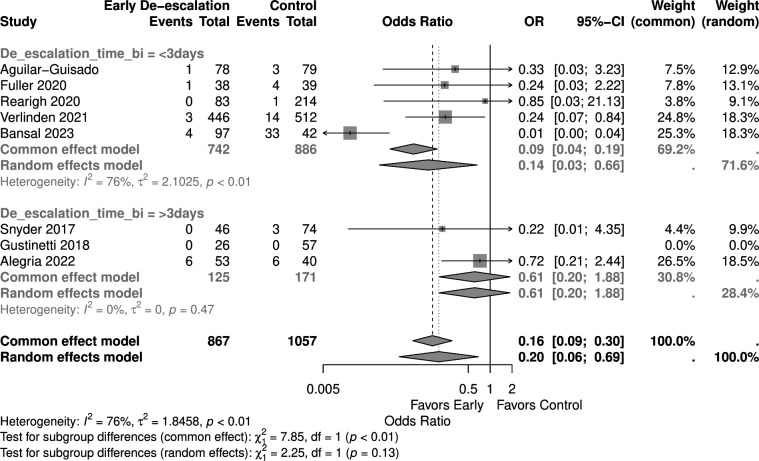
Forest plot of mortality subgroup analysis of early de-escalation time.

**Fig 11:**
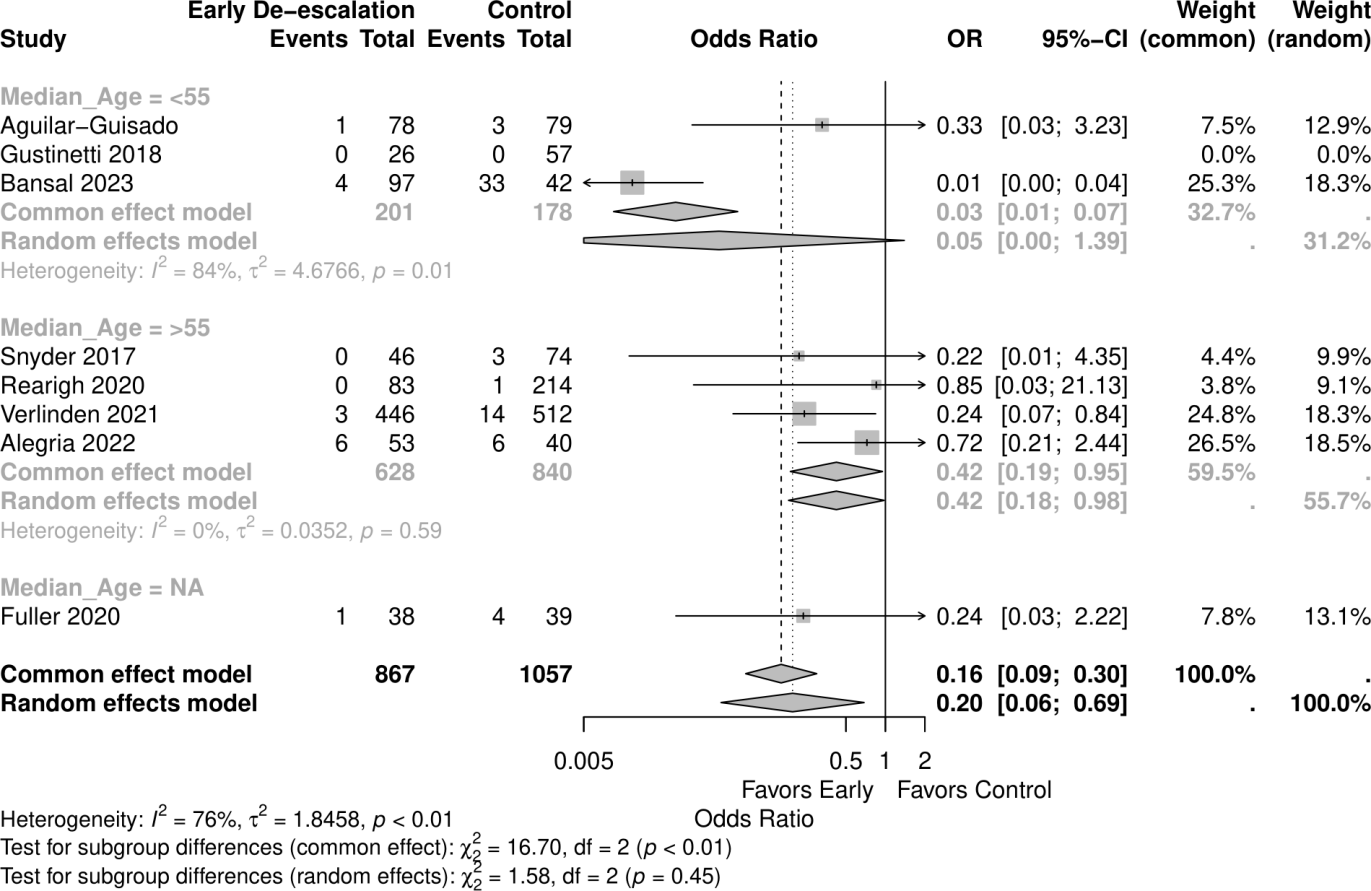
Forest plot of mortality subgroup analysis of mean age.

**Fig 12:**
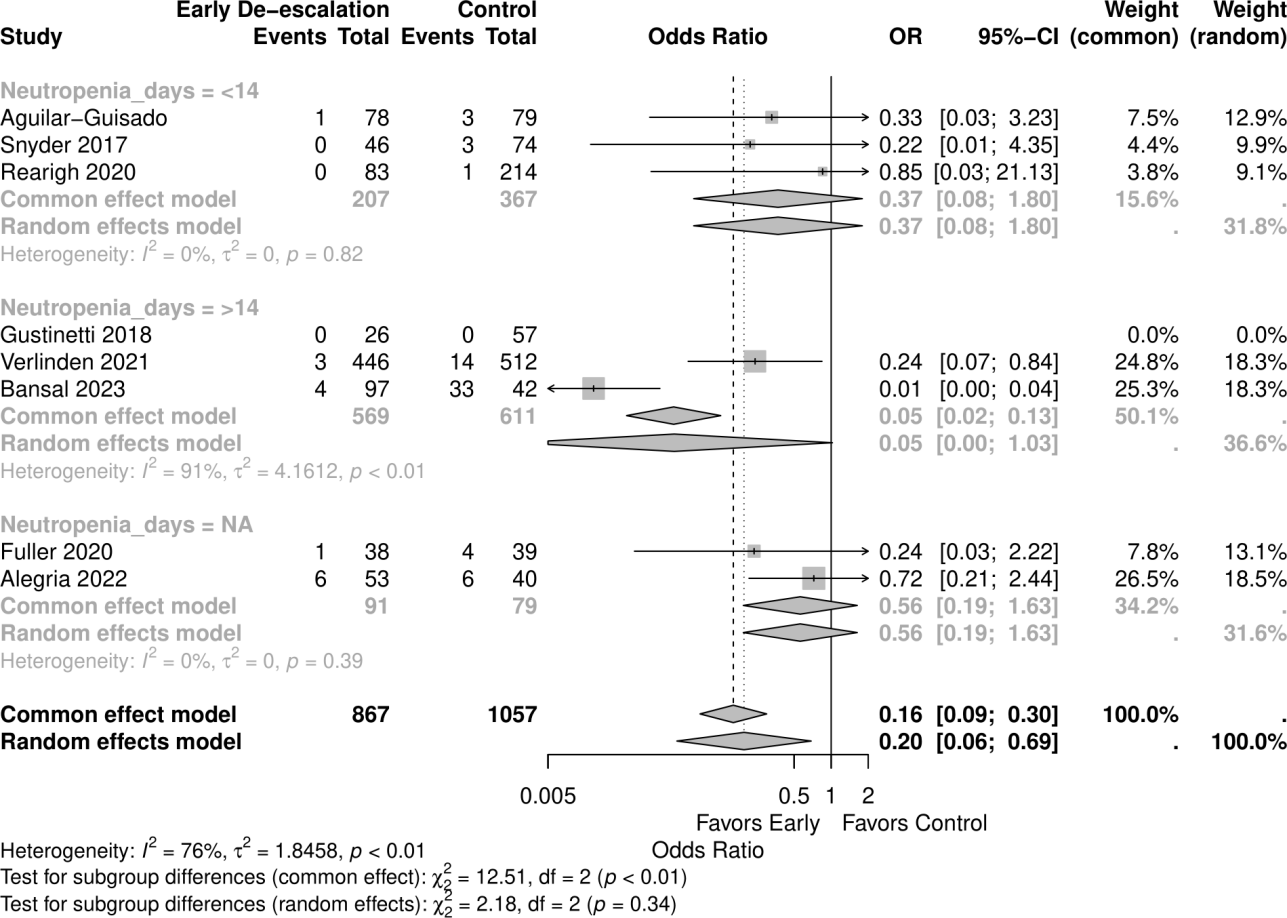
Forest plot of mortality subgroup analysis of neutropenia days.

**Fig 13:**
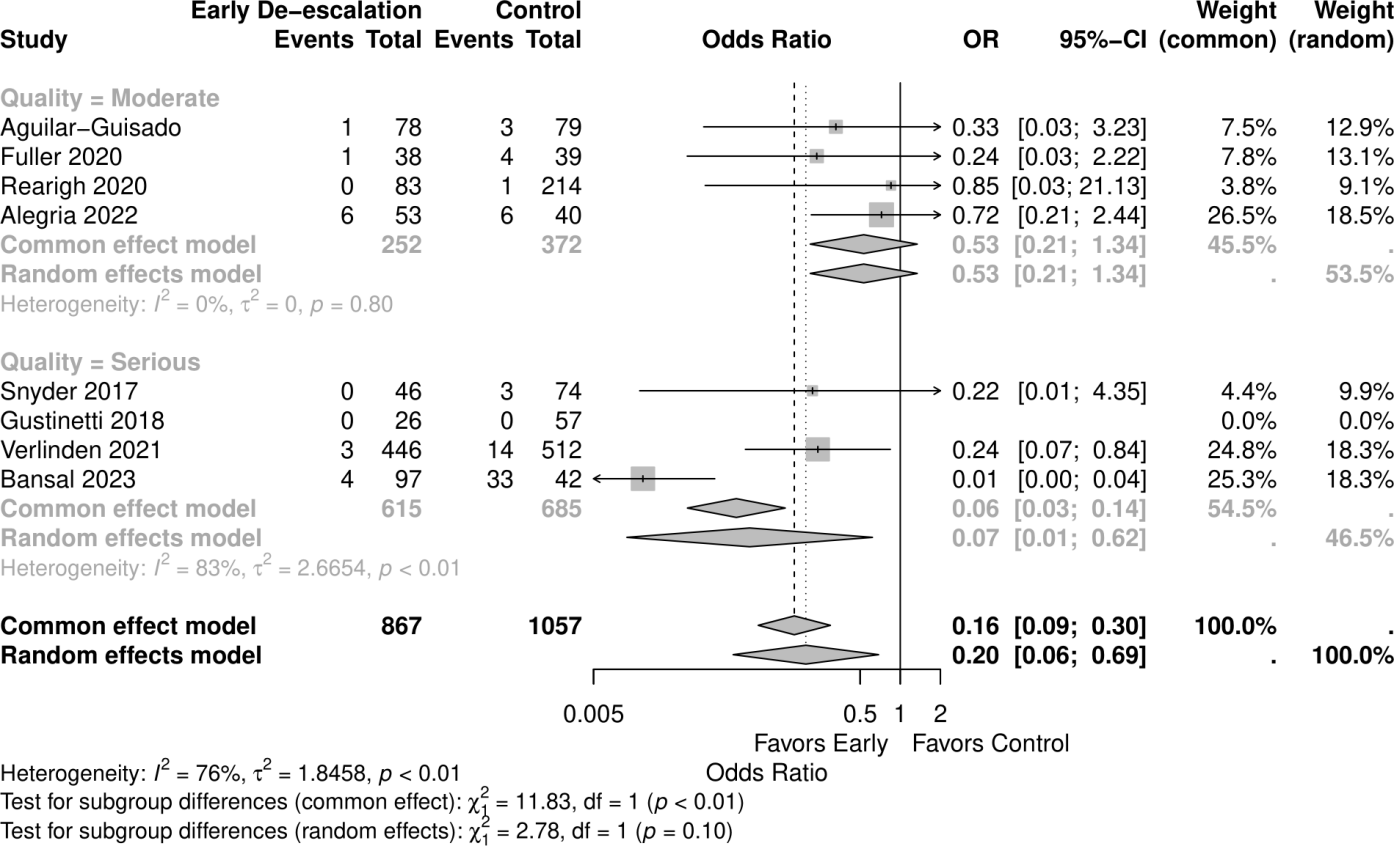
Forest plot of mortality subgroup analysis of study quality.

**Fig 14:**
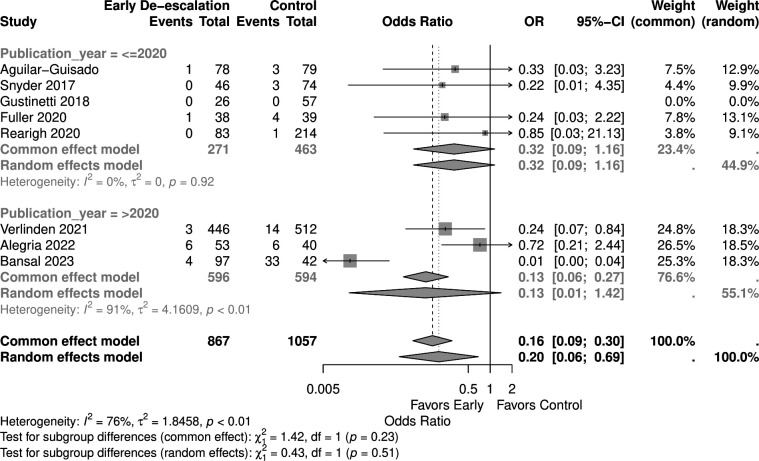
Forest plot of mortality subgroup analysis of publication year.

Studies with a higher proportion of AML patients (>30%) showed no significant mortality difference (OR 0.05, 95% CI 0.00–1.03) in the random effect model, similar to those with <30% AML patients (OR 0.53, 95% CI 0.20–1.38) (Fig. 9). Early de-escalation within 3 days was associated with significantly lower mortality risk (OR 0.14, 95% CI 0.03–0.66), but not de-escalation after 3 days (OR 0.61, 95% CI 0.20–1.88) (Fig. 10). When stratified by median age, studies with patients more than 55 years showed a significantly lower mortality risk with early de-escalation (OR 0.42, 95% CI 0.18–0.98), while those less than 55 years showed no significant difference in the random effect model (OR 0.05, 95% CI 0.00–1.39) (Fig. 11). Neutropenia duration did not significantly impact mortality outcomes, with no significant differences observed in studies reporting >14 days (OR 0.05, 95% CI 0.00–1.03) or <14 days (OR 0.37, 95% CI 0.08–1.80) of neutropenia (Fig. 12). Regarding study quality, a significant mortality benefit was observed in studies rated as serious quality (OR 0.07, 95% CI 0.01–0.62), while those of moderate quality showed no significant difference (OR 0.53, 95% CI 0.21–1.34), with a significant subgroup difference noted (Fig. 13). Publication year analysis revealed that studies published before 2020 prior to the COVID-19 pandemic showed no significant mortality difference (OR 0.32, 95% CI 0.09–1.16), while studies published after 2020 also showed no significant mortality difference (OR 0.13, 95% CI 0.01–1.42) (Fig. 14).

Subgroup analysis of the secondary outcomes based on study quality was also performed. The results revealed that, in the serious quality subgroup, early de-escalation was associated with a significantly higher risk of bacteremia (OR 2.06, 95% CI 1.59–2.65) and a significant reduction in the risk of recurrent fever (OR 0.74, 95% CI 0.58–0.95). In contrast, in the moderate quality subgroup, early de-escalation was not associated with a significant risk of all four secondary outcomes.

## DISCUSSION

Our meta-analysis indicated that early de-escalation of BSA in FN patients with hematological malignancies after treatment significantly reduced mortality risk compared to those with continuation of the BSA. Meanwhile, there was no significant impact on adverse events of infection-related ICU admissions, bacteremia, recurrent fever, and CDI.

In our included studies, Fuller et al. (30), Rearigh et al. (31), Snyder et al. (32), and Alegria et al. (35) revealed no significant difference in mortality risk reduction. However, Verlinden et al. (33) and Bansal et al. (36) revealed a significant reduction in mortality in the early de-escalation groups. Most of the previous studies were small, with only Verlinden et al. reaching nearly a thousand patients (33). Further sensitivity analysis excluding Bansal et al. (36) due to its result being identified as an outlier still revealed a significantly decreased mortality risk. In our study, RIS has not been reached in TSA, but the cumulative Z-curve has crossed the lower boundary, indicating a favor of the reduction in mortality risk with the intervention of early de-escalation of antibiotics. To our knowledge, our study is the first meta-analysis to report a significant decrease in mortality with early de-escalation of BSA treatment in hematologic malignancy patients with FN, and the result of TSA further validates the finding.

Verlinden et al. suggested that the reduction in overall mortality was mainly from the reduction in fatal respiratory and fungal infections (33). While Bansal et al. reported only all-cause mortality, they did not distinguish between infection-related mortality and other causes of death. This lack of specificity may have led to over- or under-estimated mortality. Additionally, while most studies included patients with FN of unknown origin, the study by Bansal specifically examined patients with carbapenem-resistant Enterobacteriaceae bacteremia; this might be the reason for the high mortality incidence in their study (36). Another study has proved that carbapenem-resistant Enterobacteriaceae is related to increased mortality in patients with hematologic malignancies (39). Prolonged BSA use can contribute to the development of resistant organisms, potentially worsening outcomes. Early de-escalation of the BSA treatment might be able to prevent resistant bacteria and therefore decrease mortality. In addition, FN is not always caused by an infection. Other potential causes include chemotherapy-induced mucositis, tumor fever, transfusion-related fever, drug fever, or graft-versus-host disease. In such cases, antibiotics may not address the underlying issue and could instead lead to unnecessary harm, including antibiotic-related toxicity and the development of resistant organisms (40, 41).

A previous study has suggested that antibiotic stewardship is related to decreased mortality in critically ill patients (42). Longer antibiotic duration and broader coverage did not yield better outcomes and led to increased resistant bacteria (43). However, Tabah et al. also pointed out the difficulty of truly identifying the reason for the mortality benefit without confounding factors (42). Other studies agreed with the consensus of practicing early de-escalation to decrease resistant bacteria when it is safe in patients with hospital-acquired pneumonia and cirrhosis (44, 45).

In our secondary outcomes analysis, a difference between the random-effect model and the fixed-effect model was observed in the risk of bacteremia. The fixed-effect model should be interpreted carefully, given that the study by Verlinden et al. (33) accounted for 72.9% of the weight. In contrast, the random-effect model had a more balanced weight distribution and revealed no significant difference. Verlinden et al. suggested that the elevation of bacteremia in the intervention group was not associated with an increase in infectious complications, such as severe sepsis, septic shock, or infection-related ICU admission, which aligned with our findings.

Despite the mixed findings of the included studies, other secondary outcomes, including infection-related ICU admissions, recurrent fever, and CDI, revealed no significant difference between the groups. This implies that these outcomes might not have played a role in the mortality reduction. It is still unclear whether infection or non-infection outcomes influence mortality. Further studies are needed to determine the reason for the reduction in mortality.

Our subgroup analyses revealed a significant mortality reduction in studies involving older patients (median age over 55 years). Previous reviews have noted that older patients are often treated with polypharmacy regimens, increasing the risk of drug-drug interactions (46). Aging is associated with a decline in the functional reserve of multiple organs, which can affect pharmacokinetics and pharmacodynamics in the elderly. As a result, this subgroup might have low drug tolerability, further leading to poor adherence, increasing the risk of antimicrobial resistance or drug overexposure, resulting in adverse effects. Early de-escalation may mitigate these risks, offering a safer therapeutic approach for elderly patients by reducing possible adverse effects.

Our study has the strength of a larger sample size, but there were also several limitations. Most of the included studies were retrospective, which might be prone to bias and confounding factors. Additionally, our subgroup analysis revealed different results in mortality depending on the study quality, suggesting that further high-quality prospective or randomized high-quality studies are needed to draw more definitive conclusions toward mortality benefit. Lastly, the underlying hematologic malignancies and early de-escalation methods among the included studies varied. The heterogeneity in patient populations and de-escalation strategies complicates the process of drawing definitive conclusions. This variability might be reflected in the lack of significant differences in secondary outcomes. Despite these limitations, the most clinically relevant implication of this meta-analysis is that early de-escalation did not result in worse patient outcomes and may even provide a mortality benefit. Further studies are warranted to implement more universal guidelines regarding the strategy of early de-escalation of BSA.

In conclusion, our meta-analysis demonstrates that early de-escalation of broad-spectrum antibiotics in FN patients with hematologic malignancies significantly reduces mortality risk without increasing adverse events such as infection-related ICU admissions, bacteremia, recurrent fever, or CDI. The findings suggest that early de-escalation, particularly in older patients, may offer a safer and more effective treatment approach by reducing potential drug-related risks and preventing antimicrobial resistance. However, the heterogeneity among study designs and the retrospective nature of many included studies highlighted the need for further high-quality prospective research to validate these findings and develop standardized guidelines for early de-escalation strategies.

## Data Availability

All data generated or analyzed during this study are included in this published article and its supplemental files.

## SUPPLEMENTAL MATERIAL

Supplement 1 aac.01597-24-s0001.docx Search strategy.

Supplement 2 aac.01597-24-s0002.pdf Funnel plot of mortality.

Supplement 3 aac.01597-24-s0003.pdf Funnel plot of infection-related ICU admission.

Supplement 4 aac.01597-24-s0004.pdf Funnel plot of bacteremia.

Supplement 5 aac.01597-24-s0005.pdf Funnel plot of recurrent fever.

Supplement 6 aac.01597-24-s0006.pdf Funnel plot of CDI.

Supplement 7 aac.01597-24-s0007.pdf Forest plot of infection-related ICU admission subgroup analysis based on study quality.

Supplement 8 aac.01597-24-s0008.pdf Forest plot of bacteremia subgroup analysis based on study quality.

Supplement 9 aac.01597-24-s0009.pdf Forest plot of recurrent fever subgroup analysis based on study quality.

Supplement 10 aac.01597-24-s0010.pdf Forest plot of CDI subgroup analysis based on study quality.

ASM does not own the copyrights to Supplemental Material that may be linked to, or accessed through, an article. The authors have granted ASM a non-exclusive, world-wide license to publish the Supplemental Material files. Please contact the corresponding author directly for reuse.
