# Supplementary material for: Efficacy and safety of early antibiotic de-escalation in febrile neutropenia for patients with hematologic malignancy: a systematic review and meta-analysis
Source: Antimicrob Agents Chemother. 2025 Mar 13;69(4):e01597-24. doi: 10.1128/aac.01597-24 (PMC11963549; doi:10.1128/aac.01597-24)
Supplement: Supplement 1 — Search strategy. [file aac.01597-24-s0001.docx]

**Supplement 1 Search Strategy**

PubMed

(("Hematopoietic Stem Cell Transplantation"[Mesh] OR "Bone Marrow Transplantation"[Mesh] OR "Hematopoietic Stem Cell Transplantation"[tiab] OR "Bone Marrow Transplantation"[tiab] OR "stem cell transplant*"[tiab] OR "bone marrow transplant*"[tiab] OR HSCT[tiab] OR BMT[tiab])

OR

("Chemotherapy, Adjuvant"[Mesh] OR "chemotherapy"[tiab] OR "anticancer treatment"[tiab] OR "cytotoxic therapy"[tiab] OR "antineoplastic therapy"[tiab]))

AND

("Neutropenia"[Mesh] OR neutropeni*[tiab] OR "Febrile Neutropenia"[Mesh] OR "febrile neutropenia"[tiab] OR "neutropenic fever"[tiab] OR FN[tiab]) ) AND ("Anti-Bacterial Agents"[Mesh] OR "anti-bacterial agent*"[tiab] OR antibacterial*[tiab] OR "Antibiotic Prophylaxis"[Mesh] OR "antibiotic prophylaxis"[tiab] OR antibiotics[tiab] OR antimicrobial*[tiab] OR antibacterial*[tiab])

AND

(de-escalat*[tiab] OR discontinu*[tiab] OR "duration of therapy"[tiab] OR cessation[tiab] OR withdrawal[tiab] OR "antibiotic stewardship"[tiab])

AND

("empiric antibiotic*"[tiab] OR empirical[tiab] OR broad-spectrum[tiab] OR "broad spectrum"[tiab] OR "Standard of Care"[tiab])

**Embase:**

#1 'hematopoietic stem cell transplantation'/exp

#2 'bone marrow transplantation'/exp

#3 'chemotherapy'/exp

#4 'hematopoietic stem cell transplantation':ti,ab OR 'bone marrow transplantation':ti,ab OR 'stem cell transplant*':ti,ab OR 'bone marrow transplant*':ti,ab OR hsct:ti,ab OR bmt:ti,ab

#5 'chemotherapy':ti,ab OR 'anticancer treatment':ti,ab OR 'cytotoxic therapy':ti,ab OR 'antineoplastic therapy':ti,ab

#6 #1 OR #2 OR #3 OR #4 OR #5

#7 'neutropenia'/exp

#8 'febrile neutropenia'/exp

#9 neutropeni*:ti,ab OR 'febrile neutropenia':ti,ab OR 'neutropenic fever':ti,ab OR fn:ti,ab

#10 #7 OR #8 OR #9

#11 'antibiotic agent'/exp

#12 'anti-bacterial agent*':ti,ab OR antibacterial*:ti,ab OR 'antibiotic prophylaxis':ti,ab OR antibiotics:ti,ab OR antimicrobial*:ti,ab OR antibacterial*:ti,ab

#13 #11 OR #12

#14 'antibiotic therapy'/exp

#15 de-escalat*:ti,ab OR discontinu*:ti,ab OR 'duration of therapy':ti,ab OR cessation:ti,ab OR withdrawal:ti,ab OR 'antibiotic stewardship':ti,ab

#16 'empiric antibiotic*':ti,ab OR empirical:ti,ab OR 'broad-spectrum':ti,ab OR 'broad spectrum':ti,ab OR 'standard of care':ti,ab

#17 #6 AND #10 AND #13 AND (#14 OR #15) AND #16

**Cochrane Library:**

#1 MeSH descriptor: [Hematopoietic Stem Cell Transplantation] explode all trees

#2 MeSH descriptor: [Bone Marrow Transplantation] explode all trees

#3 MeSH descriptor: [Chemotherapy, Adjuvant] explode all trees

#4 ("hematopoietic stem cell transplantation" OR "bone marrow transplantation" OR "stem cell transplant*" OR "bone marrow transplant*" OR HSCT OR BMT OR "chemotherapy" OR "anticancer treatment" OR "cytotoxic therapy" OR "antineoplastic therapy"):ti,ab,kw

#5 #1 OR #2 OR #3 OR #4

#6 MeSH descriptor: [Neutropenia] explode all trees

#7 MeSH descriptor: [Febrile Neutropenia] explode all trees

#8 (neutropeni* OR "febrile neutropenia" OR "neutropenic fever" OR FN):ti,ab,kw

#9 #6 OR #7 OR #8

#10 MeSH descriptor: [Anti-Bacterial Agents] explode all trees

#11 ("anti-bacterial agent*" OR antibacterial* OR "antibiotic prophylaxis" OR antibiotics OR antimicrobial* OR antibacterial*):ti,ab,kw

#12 #10 OR #11

#13 (de-escalat* OR discontinu* OR "duration of therapy" OR cessation OR withdrawal OR "antibiotic stewardship"):ti,ab,kw

#14 ("empiric antibiotic*" OR empirical OR broad-spectrum OR "broad spectrum" OR "Standard of Care"):ti,ab,kw

#15 #5 AND #9 AND #12 AND #13 AND #14

Supplement 2 Funnel Plot of Mortality

Supplement 3 Funnel Plot of Infection-Related ICU Admission

Supplement 4 Funnel Plot of Bacteremia

Supplement 5 Funnel Plot of Recurrent Fever

Supplement 6 Funnel Plot of CDI

Supplement 7 Forest Plot of Infection-related ICU Admissions Subgroup Analysis Based on Study Quality

Supplement 8 Forest Plot of Bacteremia Subgroup Analysis Based on Study Quality

Supplement 9 Forest Plot of Recurrent Fever Subgroup Analysis Based on Study Quality

Supplement 10 Forest Plot of CDI Subgroup Analysis Based on Study Quality
