# Supplementary figures and images for: Efficacy and safety of early antibiotic de-escalation in febrile neutropenia for patients with hematologic malignancy: a systematic review and meta-analysis
Source: Antimicrob Agents Chemother. 2025 Mar 13;69(4):e01597-24. doi: 10.1128/aac.01597-24 (PMC11963549; doi:10.1128/aac.01597-24)

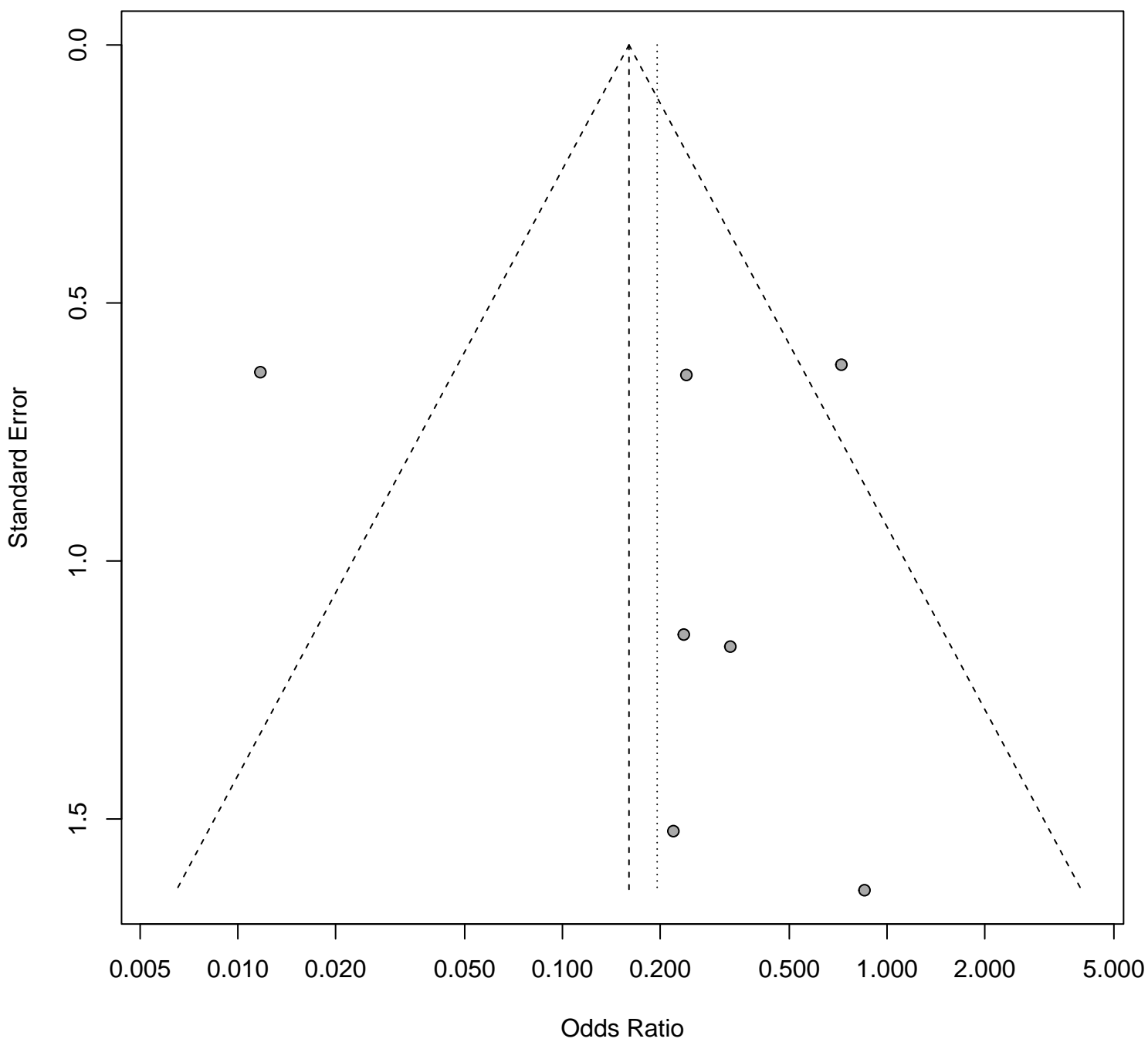

Supplement: Supplement 2 — Funnel plot of mortality. [file aac.01597-24-s0002.pdf]

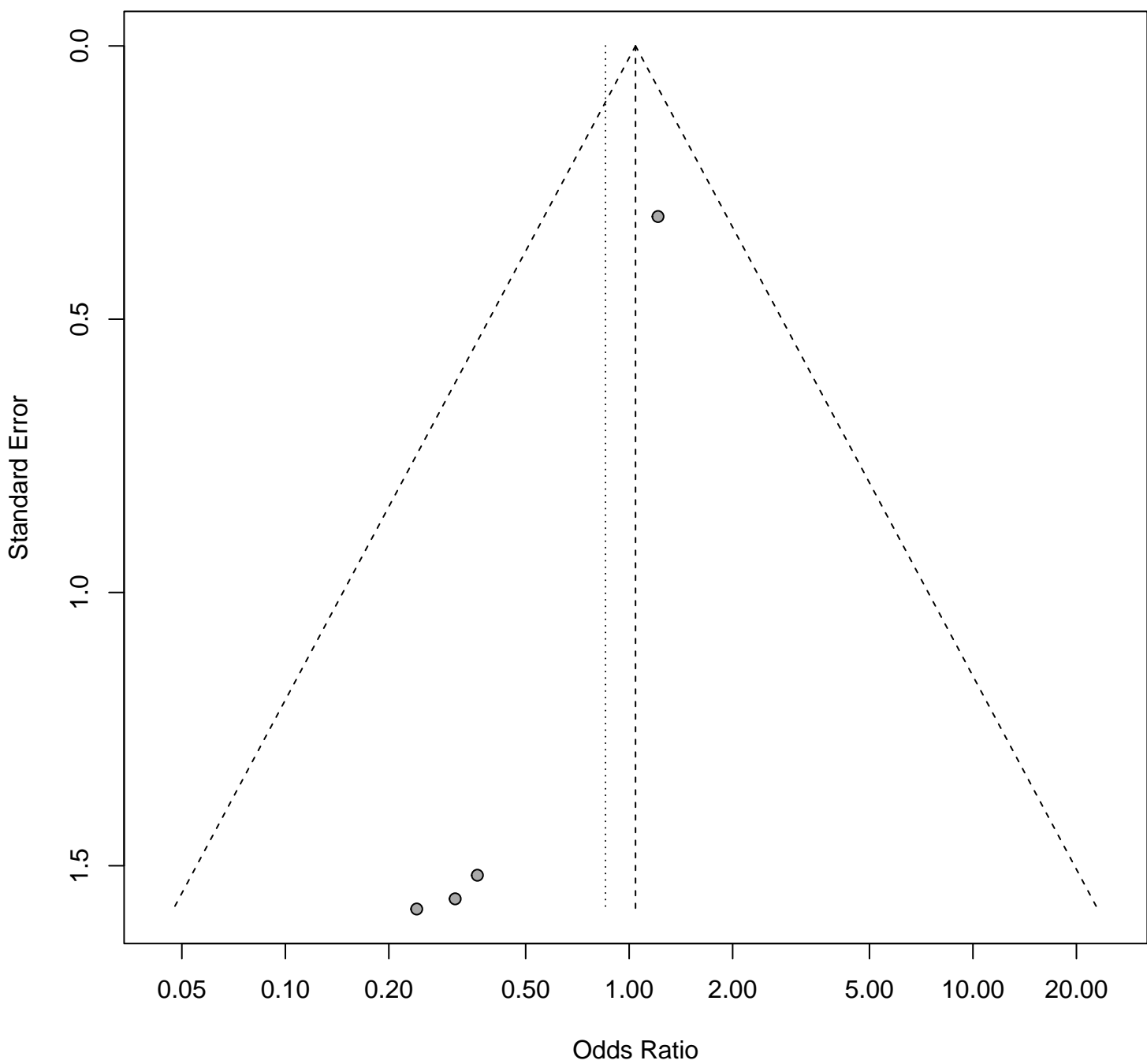

Supplement: Supplement 3 — Funnel plot of infection-related ICU admission. [file aac.01597-24-s0003.pdf]

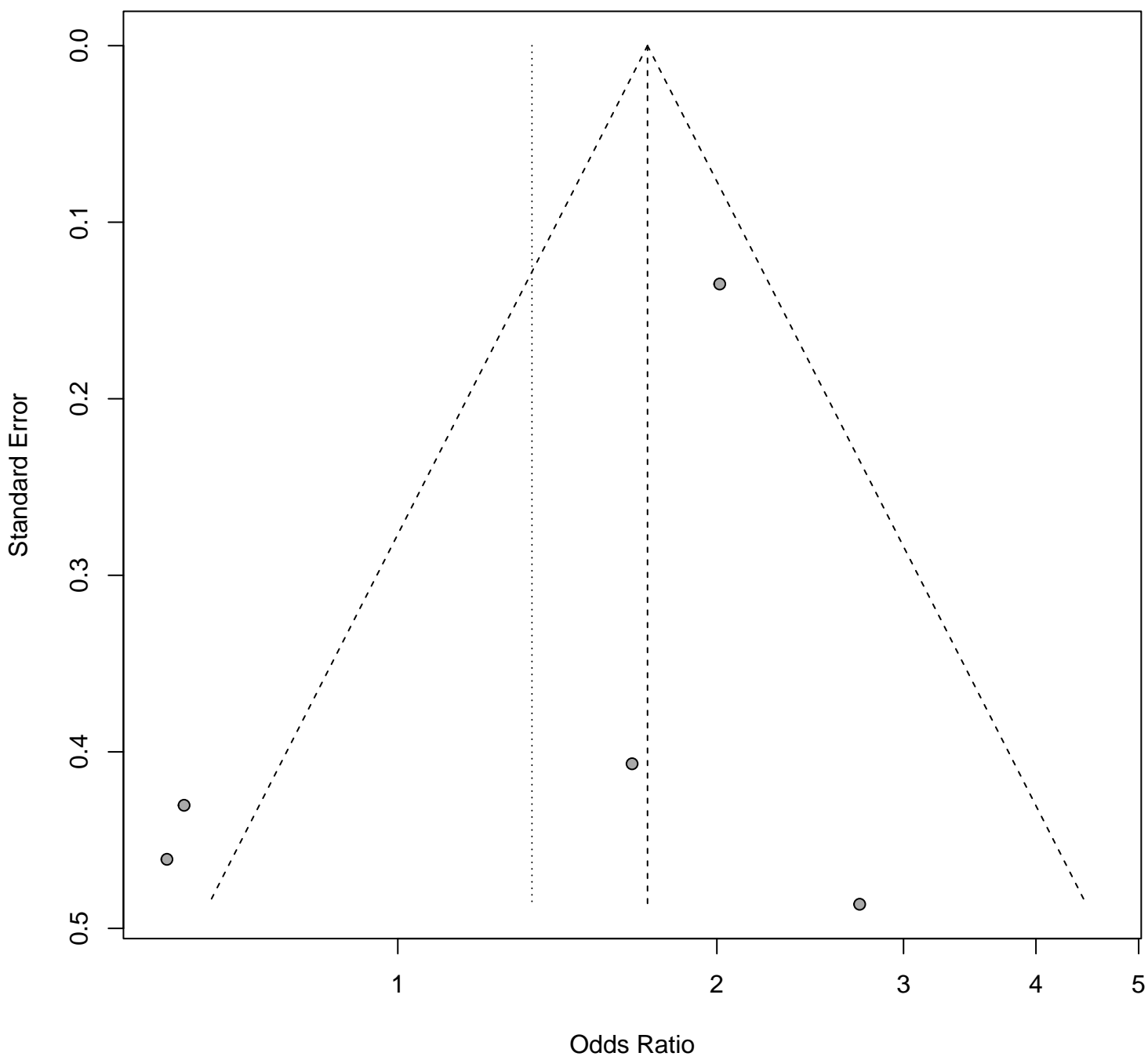

Supplement: Supplement 4 — Funnel plot of bacteremia. [file aac.01597-24-s0004.pdf]

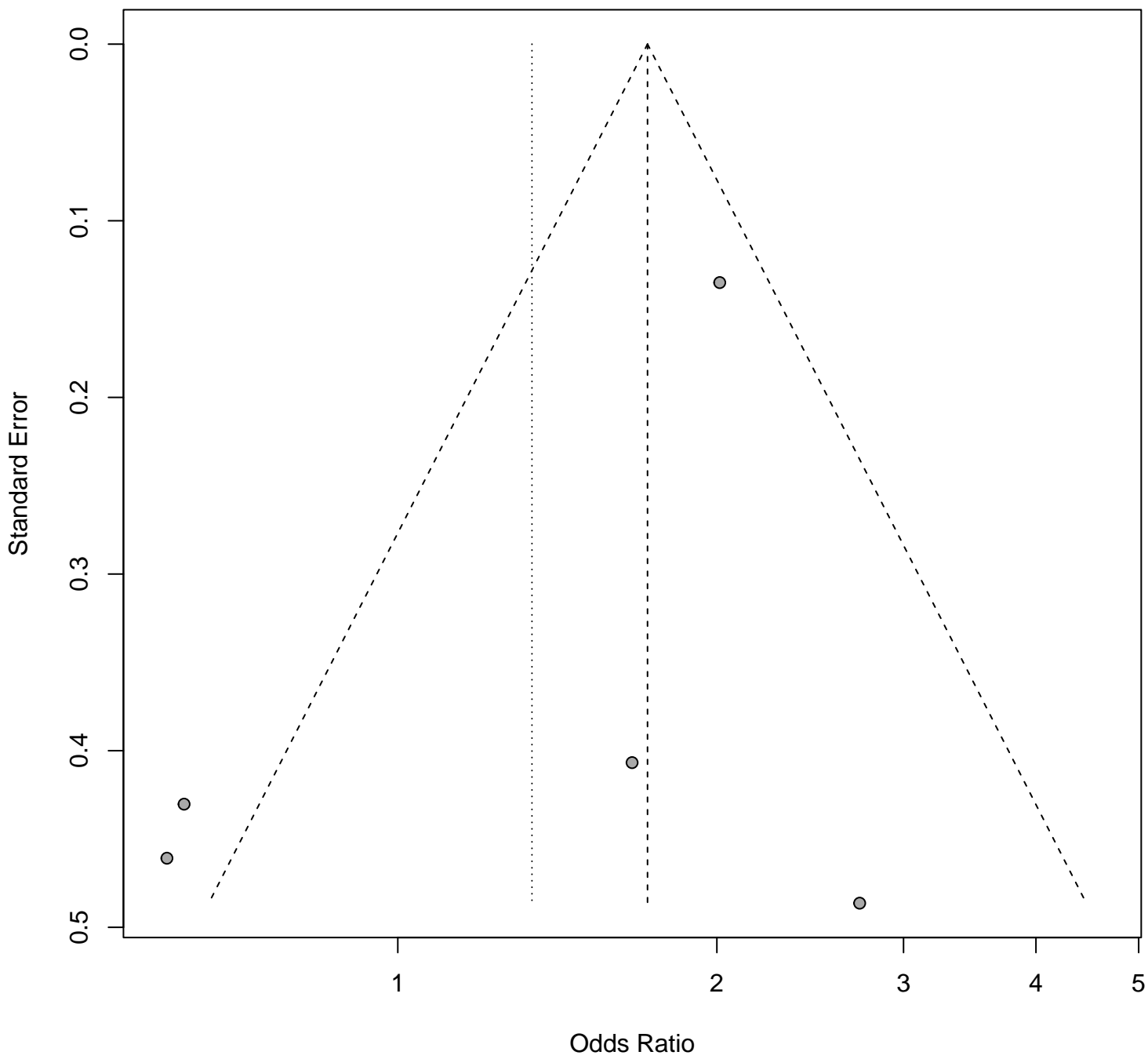

Supplement: Supplement 5 — Funnel plot of recurrent fever. [file aac.01597-24-s0005.pdf]

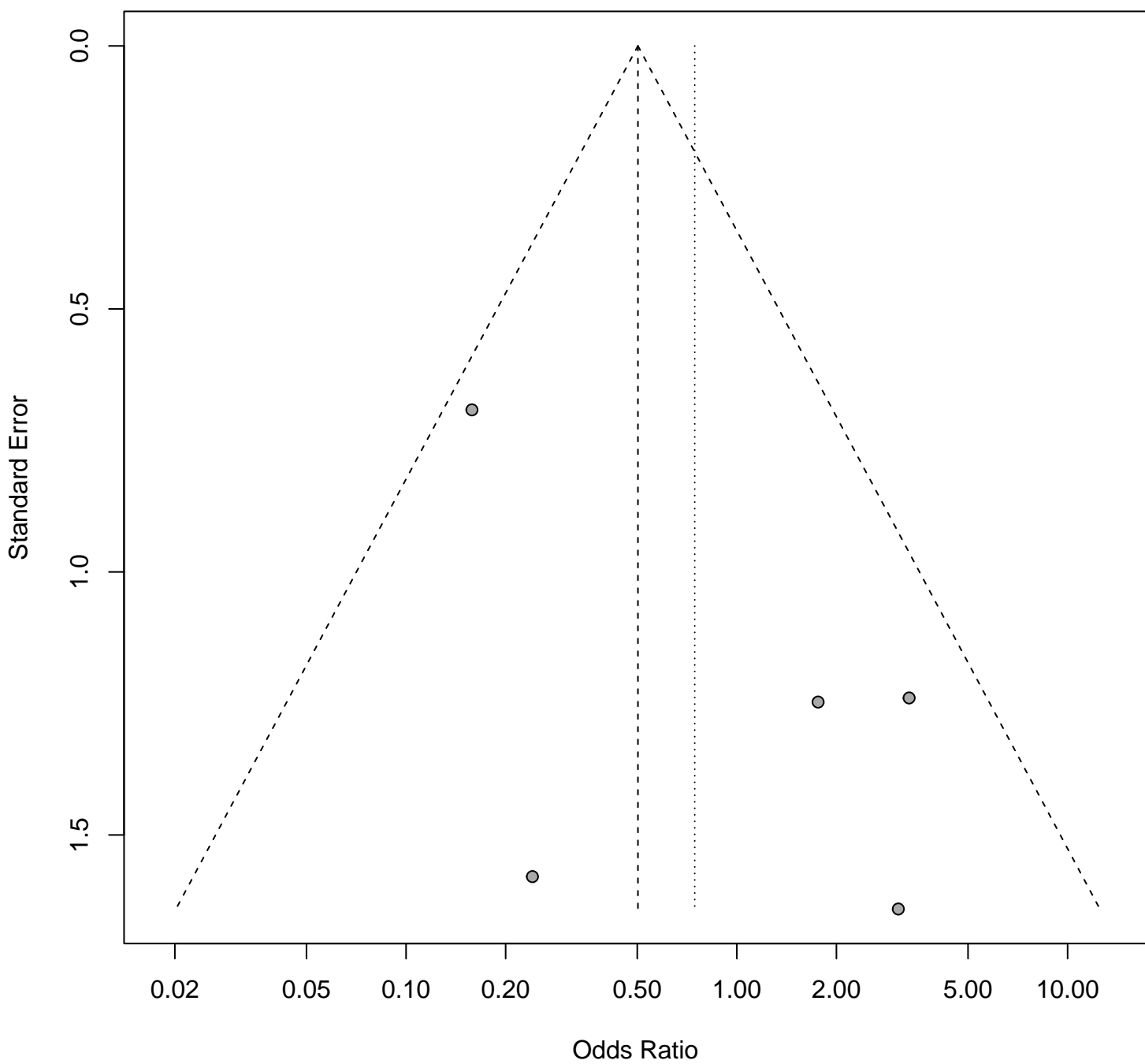

Supplement: Supplement 6 — Funnel plot of CDI. [file aac.01597-24-s0006.pdf]
