# Supplementary material for: Efficacy and safety of early antibiotic de-escalation in febrile neutropenia for patients with hematologic malignancy: a systematic review and meta-analysis
Source: Antimicrob Agents Chemother. 2025 Mar 13;69(4):e01597-24. doi: 10.1128/aac.01597-24 (PMC11963549; doi:10.1128/aac.01597-24)
Supplement: Supplement 7 — Forest plot of infection-related ICU admission subgroup analysis based on study quality. [file aac.01597-24-s0007.pdf]

Study	Early De−escalation Events	Early De−escalation Total	Control Events	Control Total	OR	95%−CI	Weight (common)	Weight (random)
**Quality = Serious**								
Snyder 2017	0	46	2	74	0.31	[0.01; 6.64]	3.6%	8.3%
Verlinden 2021	22	446	21	512	1.21	[0.66; 2.24]	89.2%	74.7%
**Common effect model**		**492**		**586**	**1.15**	**[0.63; 2.10]**	**92.7%**	**.**
**Random effects model**					**1.15**	**[0.63; 2.10]**	**.**	**83.0%**
Heterogeneity: $I^2 = 0\%$, $\tau^2 = 0$, $p = 0.39$								
**Quality = Moderate**								
Rearigh 2020	0	83	3	214	0.36	[0.02; 7.08]	3.8%	8.8%
Ly 2021	0	19	2	25	0.24	[0.01; 5.32]	3.5%	8.2%
**Common effect model**		**102**		**239**	**0.30**	**[0.03; 2.54]**	**7.3%**	**.**
**Random effects model**					**0.30**	**[0.03; 2.54]**	**.**	**17.0%**
Heterogeneity: $I^2 = 0\%$, $\tau^2 = 0$, $p = 0.85$								
**Common effect model**		**594**		**825**	**1.04**	**[0.59; 1.86]**	**100.0%**	**.**
**Random effects model**					**0.85**	**[0.34; 2.14]**	**.**	**100.0%**

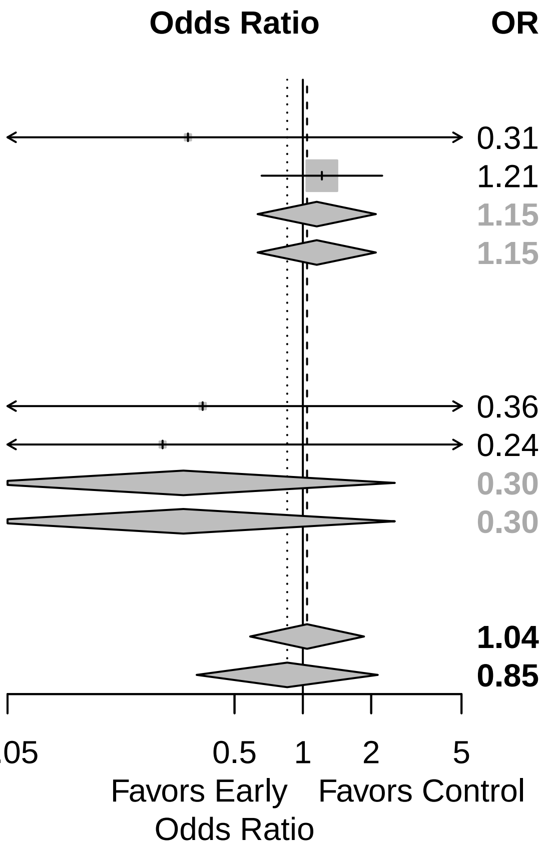

Heterogeneity: $I^2 = 0\%$, $\tau^2 = 0.1967$, $p = 0.54$
Test for subgroup differences (common effect): $\chi^2_1 = 1.42$, df = 1 ($p = 0.23$)
Test for subgroup differences (random effects): $\chi^2_1 = 1.42$, df = 1 ($p = 0.23$)
