# Supplementary material for: Efficacy and safety of early antibiotic de-escalation in febrile neutropenia for patients with hematologic malignancy: a systematic review and meta-analysis
Source: Antimicrob Agents Chemother. 2025 Mar 13;69(4):e01597-24. doi: 10.1128/aac.01597-24 (PMC11963549; doi:10.1128/aac.01597-24)
Supplement: Supplement 8 — Forest plot of bacteremia subgroup analysis based on study quality. [file aac.01597-24-s0008.pdf]

Study	Early De−escalation Events	Early De−escalation Total	Control Events	Control Total	OR	95%−CI	Weight (common)	Weight (random)
**Quality = Moderate**								
Aguilar−Guisado 2017	9	78	14	79	0.61	[0.25; 1.49]	6.3%	17.3%
Rearigh 2020	11	83	18	214	1.66	[0.75; 3.69]	8.0%	19.2%
Alegria 2022	18	53	18	40	0.63	[0.27; 1.46]	7.2%	18.3%
**Common effect model**		**214**		**333**	**0.89**	**[0.55; 1.46]**	**21.5%**	**.**
**Random effects model**					**0.88**	**[0.45; 1.71]**	**.**	**54.8%**
Heterogeneity: $I^2 = 46\%$, $\tau^2 = 0.1615$, $p = 0.16$								
**Quality = Serious**								
Gustinetti 2018	15	26	19	57	2.73	[1.05; 7.07]	5.6%	16.5%
Verlinden 2021	209	446	156	512	2.01	[1.54; 2.62]	72.9%	28.6%
**Common effect model**		**472**		**569**	**2.06**	**[1.59; 2.65]**	**78.5%**	**.**
**Random effects model**					**2.06**	**[1.59; 2.65]**	**.**	**45.2%**
Heterogeneity: $I^2 = 0\%$, $\tau^2 = 0$, $p = 0.55$								
**Common effect model**		**686**		**902**	**1.72**	**[1.37; 2.16]**	**100.0%**	**.**
**Random effects model**					**1.34**	**[0.76; 2.37]**	**.**	**100.0%**

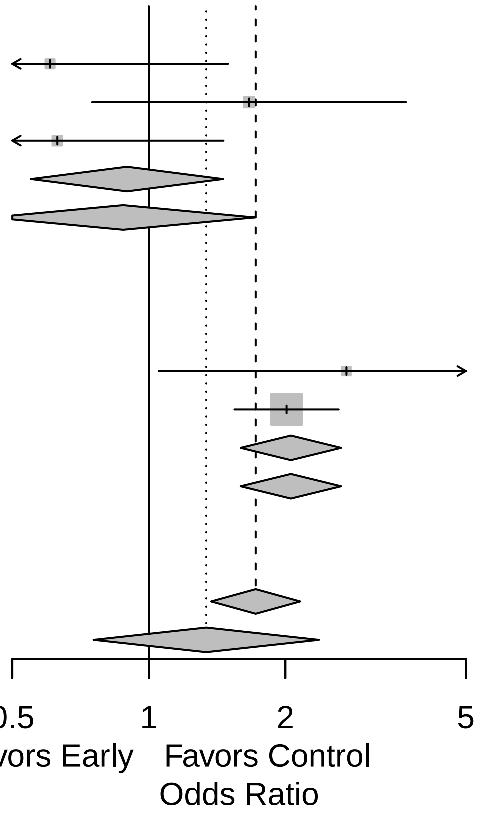

Heterogeneity: $I^2 = 69\%$, $\tau^2 = 0.2792$, $p = 0.01$

Test for subgroup differences (common effect): $\chi^2_1 = 8.78$, df = 1 ($p < 0.01$)

Test for subgroup differences (random effects): $\chi^2_1 = 5.45$, df = 1 ($p = 0.02$)
