# Supplementary material for: Efficacy and safety of early antibiotic de-escalation in febrile neutropenia for patients with hematologic malignancy: a systematic review and meta-analysis
Source: Antimicrob Agents Chemother. 2025 Mar 13;69(4):e01597-24. doi: 10.1128/aac.01597-24 (PMC11963549; doi:10.1128/aac.01597-24)
Supplement: Supplement 9 — Forest plot of recurrent fever subgroup analysis based on study quality. [file aac.01597-24-s0009.pdf]

Study	Early De−escalation Events	Early De−escalation Total	Control Events	Control Total	OR	95%−CI	Weight (common)	Weight (random)
**Quality = Moderate**								
Aguilar−Guisado 2017	11	78	14	79	0.76	[0.32; 1.80]	6.5%	14.5%
Fuller 2020	2	38	3	39	0.67	[0.11; 4.23]	1.4%	5.1%
Rearigh 2020	15	83	15	214	2.93	[1.36; 6.30]	8.2%	16.3%
Ly 2021	4	19	9	25	0.47	[0.12; 1.87]	2.6%	8.1%
**Common effect model**		**218**		**357**	**1.28**	**[0.77; 2.12]**	**18.8%**	**.**
**Random effects model**					**1.04**	**[0.42; 2.58]**	**.**	**43.9%**
Heterogeneity: $I^2 = 64\%$, $\tau^2 = 0.4999$, $p = 0.04$								
**Quality = Serious**								
Snyder 2017	7	46	14	74	0.77	[0.29; 2.08]	4.9%	12.4%
Gustinetti 2018	8	26	23	57	0.66	[0.24; 1.76]	5.0%	12.5%
Petteys 2019	1	24	6	83	0.56	[0.06; 4.88]	1.0%	3.9%
Verlinden 2021	155	446	213	512	0.75	[0.58; 0.97]	70.3%	27.4%
**Common effect model**		**542**		**726**	**0.74**	**[0.58; 0.95]**	**81.2%**	**.**
**Random effects model**					**0.74**	**[0.58; 0.95]**	**.**	**56.1%**
Heterogeneity: $I^2 = 0\%$, $\tau^2 = 0$, $p = 0.99$								
**Common effect model**		**760**		**1083**	**0.82**	**[0.66; 1.02]**	**100.0%**	**.**
**Random effects model**					**0.88**	**[0.56; 1.38]**	**.**	**100.0%**

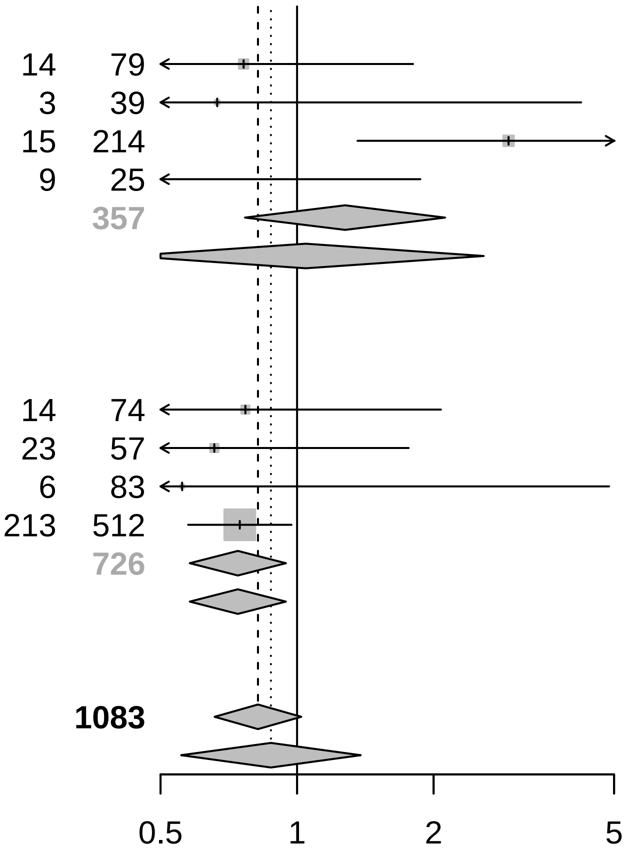

Heterogeneity: $I^2 = 42\%$, $\tau^2 = 0.1797$, $p = 0.10$
Test for subgroup differences (common effect): $\chi^2_1 = 3.58$, df = 1 ($p = 0.06$)
Test for subgroup differences (random effects): $\chi^2_1 = 0.52$, df = 1 ($p = 0.47$)
