# Supplementary material for: Efficacy and safety of early antibiotic de-escalation in febrile neutropenia for patients with hematologic malignancy: a systematic review and meta-analysis
Source: Antimicrob Agents Chemother. 2025 Mar 13;69(4):e01597-24. doi: 10.1128/aac.01597-24 (PMC11963549; doi:10.1128/aac.01597-24)
Supplement: Supplement 10 — Forest plot of CDI subgroup analysis based on study quality. [file aac.01597-24-s0010.pdf]

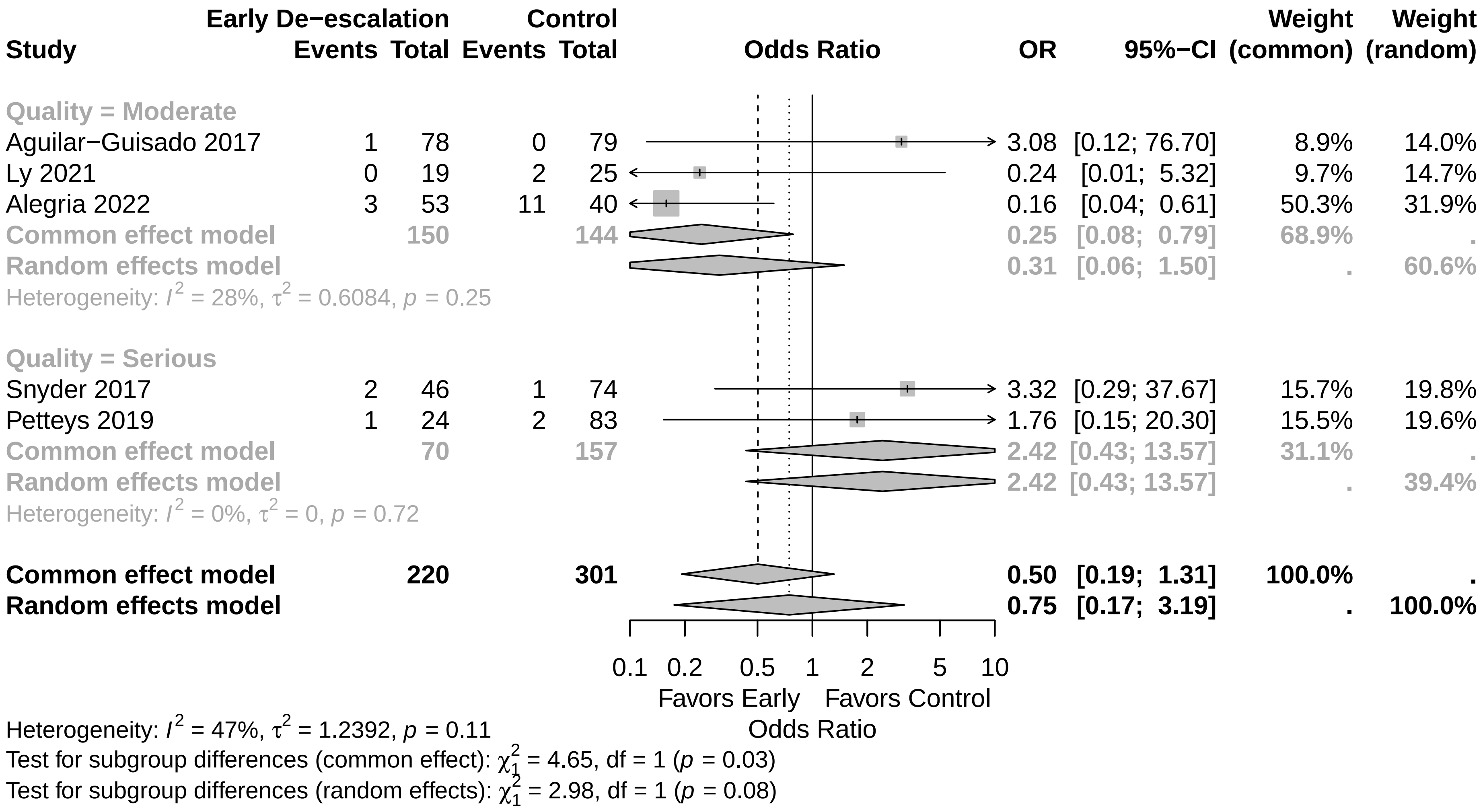
Early De−escalation
Control
Weight
Weight
Study
Events Total
Events Total
Odds Ratio
OR
95%−CI
(common)
(random)
Quality = Moderate
Aguilar−Guisado 2017 1 78 0 79 3.08 [0.12; 76.70] 8.9% 14.0%
Ly 2021 0 19 2 25 0.24 [0.01; 5.32] 9.7% 14.7%
Alegria 2022 3 53 11 40 0.16 [0.04; 0.61] 50.3% 31.9%
Common effect model 150 144 0.25 [0.08; 0.79] 68.9% .
Random effects model 0.31 [0.06; 1.50] . 60.6%
Heterogeneity: I² = 28%, τ² = 0.6084, p = 0.25
Quality = Serious
Snyder 2017 2 46 1 74 3.32 [0.29; 37.67] 15.7% 19.8%
Petteys 2019 1 24 2 83 1.76 [0.15; 20.30] 15.5% 19.6%
Common effect model 70 157 2.42 [0.43; 13.57] 31.1% .
Random effects model 2.42 [0.43; 13.57] . 39.4%
Heterogeneity: I² = 0%, τ² = 0, p = 0.72
Common effect model 220 301 0.50 [0.19; 1.31] 100.0% .
Random effects model 0.75 [0.17; 3.19] . 100.0%
0.1
0.2
0.5
1
2
5
10
Favors Early
Favors Control
Odds Ratio
Heterogeneity: I² = 47%, τ² = 1.2392, p = 0.11
Test for subgroup differences (common effect): χ²₁ = 4.65, df = 1 (p = 0.03)
Test for subgroup differences (random effects): χ²₁ = 2.98, df = 1 (p = 0.08)
